# Photocatalytic degradation activity of goji berry extract synthesized silver-loaded mesoporous zinc oxide (Ag@ZnO) nanocomposites under simulated solar light irradiation

**DOI:** 10.1038/s41598-022-14117-w

**Published:** 2022-06-15

**Authors:** Abdulrahman Ahmed Sharwani, Kannan Badri Narayanan, Mohammad Ehtisham Khan, Sung Soo Han

**Affiliations:** 1 School of Chemical Engineering, Yeungnam University, 280 Daehak-Ro, Gyeongsan, Gyeongbuk 38541 South Korea; 2 Research Institute of Cell Culture, Yeungnam University, 280 Daehak-Ro, Gyeongsan, Gyeongbuk 38541 South Korea; 3 Department of Chemical Engineering Technology, College of Applied Industrial Technology (CAIT), Jazan University, Jazan, 45971 Saudi Arabia

**Keywords:** Environmental sciences, Nanoscience and technology

## Abstract

Different approaches have been developed for the synthesis of various nanostructured materials with unique morphologies. This study demonstrated the photocatalytic and antimicrobial abilities of silver-loaded zinc oxide nanocomposites (Ag@ZnO NCs). Initially, ZnO with a unique mesoporous ellipsoidal morphology in the size range of 0.59 ± 0.11 × 0.33 ± 0.09 µm (length × width) was synthesized using aqueous precipitation in a mild hydrothermal condition (80 °C) with the aqueous fruit extract of goji berry (GB) (as an additive) and calcined in air at 200 °C/2 h and 250 °C/3 h. Powder X-ray diffraction (XRD) revealed the formation of a hexagonal phase of the wurtzite (WZ) structure. The average crystallite size of ZnO was 23.74 ± 4.9 nm as calculated using Debye–Scherrer’s equation. It also possesses higher thermal stability with the surface area, pore volume, and pore size of 11.77 m^2^/g, 0.027 cm^3^/g, and 9.52 nm, respectively. Furthermore, different mesoporous Ag@ZnO NCs loaded with face-centered cubic (fcc) silver nanoparticles (Ag NPs) in the range of 90–160 nm were synthesized by GB extract as a reducing and capping agent on the surface of ZnO after calcination in air. The immobilization of Ag NPs was confirmed by XRD, X-ray photoelectron spectroscopy (XPS), field-emission scanning electron microscopy (FE-SEM), FE-transmission electron microscopy (FE-TEM), and energy-dispersive X-ray spectroscopy (EDS). It was found that Ag_0.2_@ZnO NC (0.2 wt% of Ag) showed excellent photocatalytic degradation of both methylene blue (MB) (cationic) and congo red (CR) (anionic) dyes under simulated solar irradiation. The photocatalytic degradation of 99.3 ± 0.35% MB and 98.5 ± 1.3% CR occurred in 90 and 55 min, respectively, at room temperature by Ag_0.2_@ZnO NC. Besides, these NCs also showed broad-spectrum antibacterial activity against both Gram-positive and Gram-negative bacteria. The mechanistic concept of generating reactive oxygen species (ROS) by electron and hole charge (e‾/h^+^) carriers seems to be responsible for the photocatalytic degradation of commercial dyes and antibacterial activities by Ag@ZnO NCs. Thus, these silver-loaded mesoporous ellipsoidal ZnO NCs are promising candidates as photocatalysts for industrial/wastewater treatment as well as in antimicrobial therapeutics.

## Introduction

Dyeing and textile processing plants discharge different dyes directly as wastewater into the surrounding environment contaminating the water resources. In such cases, various treatment methods, such as adsorption and/or advanced oxidation processes (AOPs), in addition to the coagulation–flocculation process or activated sludge treatment has been used to treat wastewater. Adsorption is an effective physicochemical method to remove dyes and organics from wastewater by adsorbent without degradation of dyes^1,2^. However, AOPs are widely used technology to remove any organic recalcitrant contaminants/pollutants in wastewater. Generally, AOPs such as Fenton-like processes, ozonation, sonolysis, and photocatalysis are used for the remediation treatments in the aqueous medium^3,4^. These AOPs utilize strong oxidants, such as hydroxyl and superoxide anion radicals for the remediation of pollutants and antimicrobial activity. However, the drawbacks of AOPs are mainly because of their expensive energy sources such as ultraviolet (UV) light or reagents such as ozone and hydrogen peroxide. Thus, the use of solar irradiation as a natural energy source in photocatalysis can reduce the cost of remediation. Photocatalysis is a cost-effective, eco-friendly, and sustainable catalytic process involving light energy as a renewable energy source to activate photocatalysts such as metal or semiconductor nanoparticles to degrade various environmentally hazardous pollutants^5^. It is a surface phenomenon that happens mainly on the catalyst's surface. The photocatalytic efficiency of any nanomaterials is based on their interactions with light, the generation of electron–hole (e‾/h^+^) pairs, and efficient charge separation. In the recent past, researchers have used several photocatalysts to degrade several hazardous industrial dyes and pollutants. The dyes are classified into cationic and anionic dyes that can be dissociated into positively and negatively charged ions, respectively, in an aqueous solution^6^. The cationic dyes such as methylene blue (MB), rhodamine B (Rhb), crystal violet (CV), rhodamine 6G (Rh6G), and malachite green (MG), and anionic dyes such as methyl orange (MO), acid orange 7 (AO7), phenol red (PR), eosin Y (EY), congo red (CR), and rose Bengal (RB) are widely used in textile industries and discharged as industrial effluents^7,8^. Other organic pollutants include glyphosate, carbofuran, picloram, fluometuron, aniline, methamodiphos, trichlorfon, turbophos, trichlopyr, erioglaucine, tebuthioron, and propham^9^. Using photocatalysis, both dyes and other organic pollutants can be mineralized to carbon dioxide and water without any secondary hazardous products^10,11^.

Metal and semiconductor nanoparticles exhibit unique optoelectronic properties depending on their size and shape for the photoinduced catalytic reactions^12^. Semiconductors such as zinc oxide (ZnO) and titania (TiO_2_) can act as photocatalysts. Among different semiconductors, ZnO is one of the most common biocompatible transition metal oxide semiconductors of the II–VI semiconductor group used. The low photocatalytic activity of bulk ZnO is due to its quick recombination of charge carriers and comparatively low charge separation. However, ZnO nanoparticles (NPs) with hexagonal wurtzite crystal structures exhibit good photoexcitation stability and high electron mobility. Besides, the nanostructured ZnO with a wide bandgap of 3.37 eV limits the photocatalytic activity only to the UV region and cannot exhibit any activity upon visible light irradiation^13^. The solar spectrum comprises about 4% energy from UV radiation and about 50% energy from visible light. Therefore, the materials which can utilize visible light can be developed to gain the most profit from the natural renewable solar power^14^. This can be done by doping noble metals, metal oxides, metal sulfides, or polymers in the preparation of semiconductor nanocomposites can enhance the absorption in the visible range^15^. Coupling or doping of semiconductors with metal nanoparticles has been reported to enhance photocatalytic efficiency^16^. In addition, the synthesis of nanomaterials with metal nanoparticles of silver, gold, iron, copper, ruthenium, and palladium on the surfaces of metallic oxides has significant applications in the diverse fields of biosensing, photovoltaics, energy storage, and optics along with catalysis^17^. For instance, semiconductor metal oxide nanoparticles of ZnO NPs exhibit promising applications in photocatalysis, heterogeneous catalysis, and antimicrobial therapy^18,19^. The UV light-driven photocatalytic activity of ZnO NPs is due to their wide bandgap and high exciton binding energy (60 meV). It is also known to generate reactive oxygen species (ROS), which enhances photocatalytic and antibacterial activities, and photodynamic therapy for biomedical applications^20^. The extent of ROS production and the cytotoxicity of ZnO NPs are enhanced by the interaction with cellular components and the release of zinc cations (Zn^2+^). Similarly, metal nanoparticles such as silver nanoparticles (Ag NPs) can induce oxidative stress and cellular toxicity by producing ROS species. The surface plasmon resonance (SPR) of Ag NPs can give visible light photocatalysis to Ag/ZnO nanocomposites. The nanocomposite composed of ZnO and Ag can generate more ROS and provide a cumulative effect on photocatalysis, antibacterial and anticancer activities^21^.

Generally, plant/fruit extracts are used as an eco-friendly sustainable material for the low-cost synthesis of various metal and semiconductor nanocomposites^22^. Demissie, et al*.*^18^ synthesized ZnO NPs using *Lippia* *adoensis* “Koseret” leaf extract and evaluated their antibacterial activity against both Gram-positive (*Staphylococcus* *aureus* and *Enterococcus* *faecalis*) and Gram-negative (*Escherichia* *coli* and *Klebsiella* *pneumonia*) bacteria. Similarly, using the aqueous extract of wolfberry fruit extract, Dong and colleagues synthesized highly crystalline spherical Ag NPs in the range between 3 and 15 nm^23^. Recently, Chauhan et al.^24^ employed a facile route to synthesize both ZnO and Ag-doped ZnO using the leaf extract of *Cannabis* *sativa* as a reducing and stabilizing agent. These nanoparticles were demonstrated for the photocatalytic degradation of industrial dyes (congo red and methyl orange) and antimicrobial activity. The photocatalytic and antimicrobial activities of nanocomposites are strongly governed by their morphology. Different morphologies of ZnO can be synthesized by the aqueous precipitation method by simply varying the precipitation conditions, such as the concentration of zinc ions, precipitating agent, temperature, pH, and aging time. The combination of various conditions has been reported to change the morphology^25,26^. Several additives such as polymers (polyacrylamide (PAM), poly(vinyl alcohol) (PVA), poly(ethylene glycol) (PEG), polyvinylpyrrolidone (PVP), and hydroxypropyl methylcellulose (HPMC)) and surfactant (sodium dodecyl sulfate (SDS)) have been employed to obtain a variety of ZnO morphologies including spherical, rod-like, elongated, ring-like, sheet-like, disk-like, and hexagonal prismatic structures with different dimensions^27–29^. Eco-friendly and sustainable materials of plant/fruit extracts as additives can also direct the formation of different nanostructured morphologies of ZnO, which is an underexplored and emerging field of research. In our study, for the first time, we demonstrated the use of fruit extract as an additive for ZnO formation and as a reducing and stabilizing agent for the formation of Ag NPs on Ag@ZnO NCs. This methodology was used to synthesize unique mesoporous ellipsoidal semiconductor particles of zinc oxide (ZnO) using GB extract as an additive in a simple precipitation method, and silver nanoparticles-loaded on ZnO (Ag@ZnO NCs) to form metal/semiconductor nanocomposites using the aqueous fruit extract of goji berries (GB) as a reducing and stabilizing agent. ZnO and Ag@ZnO NCs with different silver concentrations have been evaluated for their photocatalytic activity against both anionic and cationic dyes and antibacterial activity against Gram-positive (*Staphylococcus* *aureus*) and -negative (*Escherichia* *coli*) bacteria.

## Experimental

### Materials

Silver nitrate (AgNO_3_, 99%) was purchased from Sigma-Aldrich (USA). Zinc nitrate hexahydrate (extra pure grade), ammonium hydroxide (NH_4_OH), methylene blue (MB), and congo red (CR) were purchased from Duksan Pure Chemicals Co., Ltd. (South Korea). Ampicillin sodium salt was bought from Daejung Chemicals & Metals Co., Ltd. (Siheung, South Korea). Dried goji berries (*Lycium* *barbarum* L.) were purchased from Yeongcheon medicinal herb market (Yeongcheon, South Korea). Microorganisms *Escherichia* *coli* (KCTC 2571) and *Staphylococcus* *aureus* (KCTC 3881) were obtained from the Korean Collection for Type Cultures (Jeongeup, South Korea). Muller–Hinton (MH) broth medium and agar were purchased from Becton, Dickinson, and Company (Sparks, MD, USA). Deionized water was collected using a Milli-Q direct water purification system (Merck Millipore) and used to prepare all solutions.

### Preparation of goji berry (GB) extract

The aqueous extract of goji berry (GB) (*Lycium* *barbarum* L.) fruit was done as mentioned earlier^30^. Briefly, the dried GBs were chopped into small pieces and then excellently ground into a coarse powder in a mortar pestle. The aqueous extract was prepared by heating 5.0 g of GB powder in 100 mL of deionized water taken in a 250 mL Erlenmeyer flask and allowed to boil with stirring at 100 °C for 15 min. Later, the solution was cooled to room temperature and centrifuged at 4000 rpm for 10 min, and filtered through Whatman No. 1 filter paper to obtain a clarified solution of GB extract. Finally, the aqueous GB extract was stored in the refrigerator at 4 °C for the preparation of metal nanoparticles and metal/semiconductor nanocomposites.

### Synthesis of zinc oxide (ZnO) with goji berry (GB) extract

Initially, 8.0 g zinc nitrate hexahydrate was dissolved in 100 mL deionized water and stirred at room temperature for 5 min. Then, 30 mL of freshly prepared GB extract solution was added, and the pH of the solution was adjusted to 9.0 with the dropwise addition of aqueous NH_4_OH. The resultant mixture was continuously stirred under a mild hydrothermal condition of 80 °C for 24 h. The obtained yellow precipitate was collected by centrifugation at 4000 rpm for 15 min and washed twice with deionized water. The as-prepared zinc oxide (ZnO) particles were dried in a vacuum oven at 60 °C overnight, followed by calcination at 200 °C/2 h and 250 °C /3 h in air and stored in an airtight amber vial.

### Preparation of silver-loaded zinc oxide nanocomposites (Ag@ZnO NCs)

**Figure 1:**
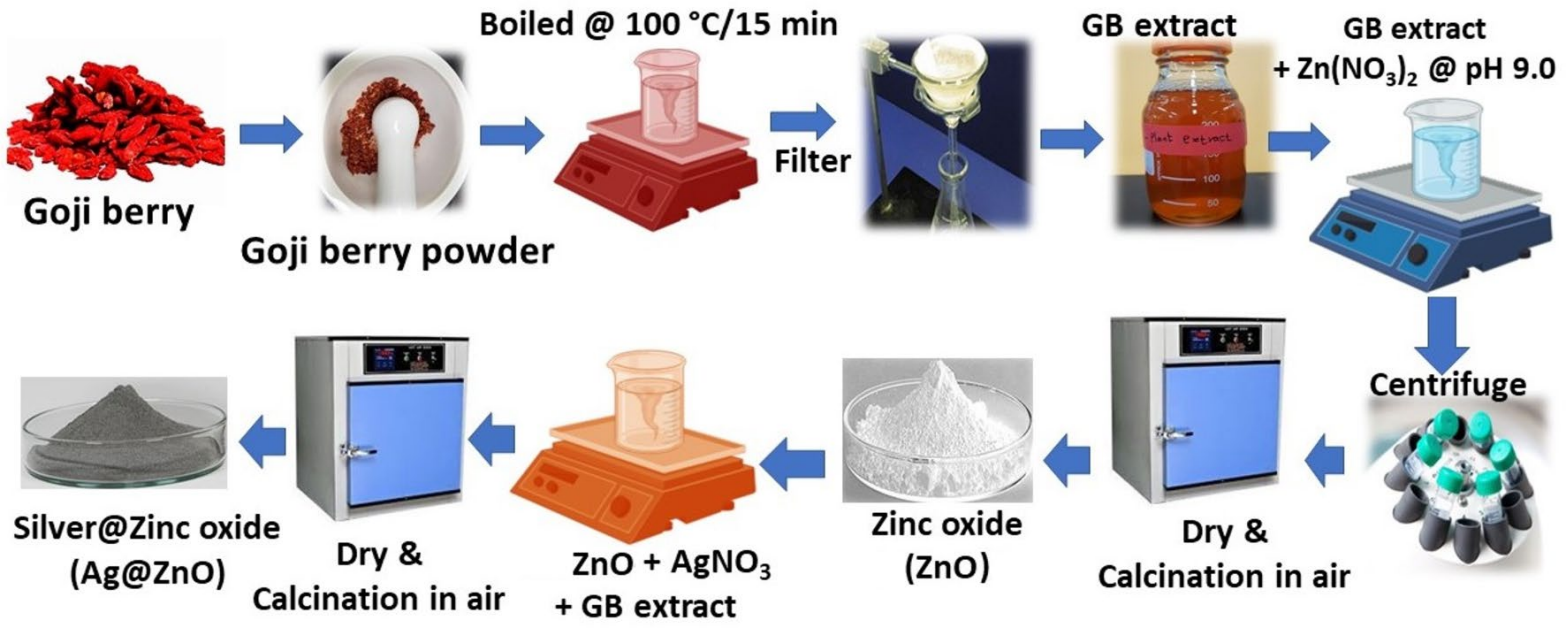
Schematic diagram of the synthesis of ZnO and Ag@ZnO NCs.

To prepare different Ag@ZnO NCs, 6.0 g of ZnO was added with varying quantities of silver (0.2%, 0.4%, and 0.8% (w/v)) in 100 mL of deionized water taken in an amber bottle. The solution was sonicated for 30 min to homogeneous the solution containing silver nitrate and ZnO. Then, 40 mL of GB extract was added dropwise with constant stirring at 60 °C for 3 h. The formed precipitate was washed three times with deionized water after centrifuging at 10,000 rpm for 20 min and dried in a hot air oven at 60 °C overnight. These dried nanocomposite powders were calcined in air at 200 °C/2 h and 250 °C/3 h and stored in an amber vial for further experiments. These nanocomposites with different silver concentrations of 0.2%, 0.4%, and 0.8% (w/v) were referred to as Ag_0.2_@ZnO, Ag_0.4_@ZnO, and Ag_0.8_@ZnO NCs, respectively. The pictorial representation of the synthesis of these metal/semiconductor nanocomposites is provided as the schematic diagram (Fig. 1).

### Characterization of ZnO and Ag@ZnO NCs

The optoelectronic properties of ZnO and different Ag@ZnO NCs were determined from the ultraviolet–visible (UV–vis) diffuse reflectance spectra (DRS) recorded using a VARIAN Cary 5000 spectrophotometer (Agilent Technologies, USA) equipped with a Praying Mantis diffuse reflectance accessory (DRA). Powder X-ray diffraction (XRD) analysis was performed to determine the crystalline structure of the nanocomposites using a PANalytical X'PertPRO MPD (Netherlands) X-ray diffractometer with Cu Kα1 radiation (0.15406 nm) and operating conditions of tube voltage 40 kV, tube current 30 mA, and scanning between 7.0° and 80.0° (2θ) at a rate of 1.2°/min. The diffraction peaks of the crystalline phases were compared with the standard compounds of the JCPDS data. The average crystallite size (D) of the samples was calculated using the Debye–Scherrer's equation: D = Kλ/βcosθ, where K is Debye–Scherrer constant (0.89), λ is the X-ray wavelength (0.15406 nm), β is the full-width at half maximum (FWHM), and θ is the diffraction angle. Fourier-transform infrared (FTIR) spectroscopy was performed using a Perkin-Elmer FTIR (Model: Spectrum 100) spectrometer in transmittance mode with the wavenumber range of 400–4000 cm^−1^. The hydrodynamic size and zeta potential of the samples were analyzed using a Zetasizer nanoparticle analyzer (Malvern Instruments Worc, UK; Model: ZS90) at 25 °C^31^.

To analyze the morphology and composition of the nanocomposites, field-emission scanning electron microscopy (FE-SEM) (Hitachi, Japan; Model: S-4200) was performed by mounting the samples on an aluminum stub and sputter-coated with platinum and analyzed with secondary electron (SE) detectors at operating voltages of 10 and 15 kV. The elemental composition was analyzed by SEM-energy dispersive X-ray spectroscopy (SEM–EDX). The shape and size of the nanocomposites were examined using an FE-transmission electron microscope (FE-TEM, FEI Tecnai G2 F20, Oregon, USA) at an accelerating voltage of 200 kV. The elemental analysis of the nanocomposites was also analyzed using the high-angle annular dark-field scanning TEM energy-dispersive X-ray spectroscopy (HAADF-STEM-EDS). The oxidation state of each element of the nanocomposite was analyzed using X-ray photoelectron spectroscopy (XPS) via a Thermo Scientific K-Alpha system with an Al Kα X-ray source and the ion source energy was between 100 V and 3.0 keV for the survey^30^. The thermal stability of nanocomposites was analyzed by thermogravimetric analysis (TGA) from room temperature to 800 °C at a heating rate of 20 °C/min in a nitrogen atmosphere. Photoluminescence (PL) spectroscopy was performed using the HORIBA Scientific Raman system and analyzed with LabSpec 6 software. A 325 nm air-cooled He-Cd laser power at 50 mW with Syncerity CCD detector was used and detected with 10 × objective in the wavelength range of 340–1050 nm. Brunauer–Emmett–Teller (BET) surface area (*S*_*BET*_), Barrett–Joyner–Halenda (BJH) pore size distribution and pore volume of samples were analyzed using a Micromeritics 3Flex adsorption analyzer (Norcross, GA, USA). The photocatalytic degradation of dyes was evaluated using a Shimadzu UV-2600 dual-beam UV–Vis spectrophotometer (Kyoto, Japan).

## Applications of ZnO and Ag@ZnO NCs

### Photocatalytic degradation of dyes

The photocatalytic degradation of dyes (MB and CR) by ZnO and Ag@ZnO NCs as photocatalysts was assessed by the decolorization of dye solutions with the initial concentrations of 10 mg/L MB or 20 mg/L CR under simulated solar light irradiation using Ultra-Vitalux lamp (300 W) (Osram GmbH). In the photocatalysis, 0.1% (w/v) of ZnO and various Ag@ZnO NCs were taken as photocatalysts and added to 100 mL of aqueous dye solutions under continuous stirring. Before simulated solar irradiation, the dye solution with photocatalyst was incubated at room temperature in the dark for 30 min to reach adsorption–desorption equilibrium. The distance between the lamp and the dye solution was kept at 10 cm, and the samples were taken periodically and centrifuged at 12,000 rpm for 10 min to remove the nanocomposites from the dye solutions. The maximum absorbance (λ_max_) of the supernatant dye solution was analyzed by a dual-beam UV–Vis spectrophotometer to quantify the concentrations of MB and CR dyes at 663 and 498 nm, respectively. The rate of degradation of dyes was calculated by the percentage of the concentration of dye that remained after a specific time over the initial dye concentration.

$${\text{Degradation }}\left( \% \right) = \left( {{\text{C}}_{0} {-}{\text{ C}}_{{\text{t}}} /{\text{C}}_{0} } \right) \times {1}00$$

where C_0_ and C_t_ are the initial and final concentrations of dyes at a reaction time (t), respectively.

### Antibacterial assay

The antibacterial activity of ZnO and Ag@ZnO NCs was tested against both Gram-negative (*E.* *coli*) and -positive (*S.* *aureus*) bacteria using the agar well diffusion method^32^. The overnight cultures of *E.* *coli* and *S.* *aureus* were obtained by inoculating the MH broth with the pure single colonies of bacteria. Later, the MH agar plates were spread-plated with pure bacterial suspensions, and the agar wells were made using a sterile cork borer with a diameter of 8 mm. Different Ag@ZnO NCs and ZnO (2 mg; 40 mg/mL) were loaded into the wells, and the plates were incubated at 37 °C for 16 h. Ampicillin (300 µg for *S.* *aureus,* and 500 µg for *E.* *coli*) was used as a positive control. The development of the zone of inhibitions (ZOIs) around the ZnO and Ag@ZnO NCs loaded wells was measured and recorded.

## Results and discussion

### Synthesis of ZnO and Ag@ZnO NCs

Generally, plant/fruit extracts have great potential in the synthesis of nanoparticles and nanocomposites. The aqueous extract of goji berries contains several phytochemicals such as phenylpropanoids, coumarins, lignans, and isoflavonoids providing natural reduction, capping, and/or stabilization moieties over the expensive chemicals to form metal nanoparticles and nanocomposites^30^. During the synthesis of ZnO by direct precipitation method with GB extract, the color of the solution changed to light yellowish and precipitated within 30 min at 80 °C (pH 9.0), indicating the formation of ZnO. ZnO nanostructure was synthesized by a simple precipitation method with the addition of ammonium hydroxide (NH_4_OH) as an oxidizer in the presence of GB extract as an additive^33^. The formation of unique morphology is perhaps the only challenge of the precipitation method. The use of several additives in precipitation aqueous solution catalyzes and functions as a morphology directing agent for the formation of unique morphology. The biomacromolecules and metabolites of GB extract direct the formation of unique ZnO nanostructures. Qi et al.^34^ reported that dextran promoted the formation of flower-like ZnO nanostructure, and positively charged homopolymer, poly-l-Lysine (PLL) as an additive was reported to catalyze the formation of ZnO formation^35^. The excess of bioorganic components attached to ZnO particles from the GB extract could have been decomposed in the calcination process in the air releasing ZnO particles with unique morphology^36,37^. Sadiq et al*.*^19^ demonstrated the synthesis of ZnO NPs using the leaf extract of *Syzygium* *cumini* (black plum). Besides secondary metabolites, plant/fruit extracts also contain many biomolecules such as proteins, polysaccharides, terpenoids, and alkaloids that could have been involved in the bioreduction and stabilization of various metal/metal oxide nanoparticles and nanocomposites^38^. When different concentrations of silver (0.2–0.8% w/v) were mixed with ZnO suspension and subsequently with GB extract, the solution color changed to light greenish, denoting the formation of Ag NPs on the surface of ZnO as Ag@ZnO NCs at 60 °C within 3 h (Fig. 1). The optical properties of the colloidal solution depend on the nanoscale morphology as well as the distance between them^39^. It has been postulated that the keto-enol tautomeric transformation of polyphenolic compounds of fruit extract such as flavonoids may release the reactive hydrogen atoms, which drive the reduction of Ag ions and enable the formation of Ag NPs^40–42^. In addition, the internal conversion of ketones to carboxylic acids in flavonoids was also likely to be involved in the reduction process of silver ions to Ag NPs^43^.

### Characterization of ZnO and Ag@ZnO NCs

**Figure 2:**
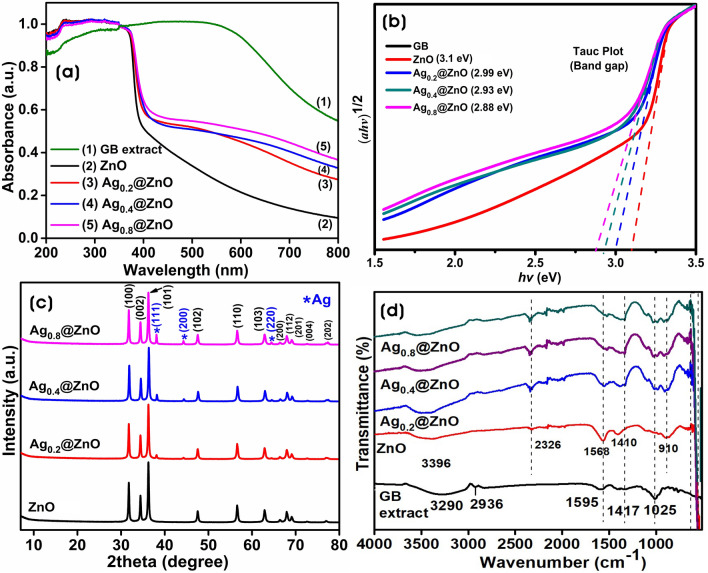
(**a**) UV–Vis DRS absorption spectra, (**b**) Tauc plots, (**c**) Powder XRD analyses, and (**d**) FTIR spectra of ZnO, Ag_0.2_@ZnO, Ag_0.4_@ZnO, and Ag_0.8_@ZnO NCs.

The optical properties of the ZnO and Ag@ZnO NCs were investigated by the UV–Vis diffuse reflectance absorption spectra. Figure 2a shows the absorption edges of ZnO and Ag@ZnO NCs in the UV region with a band edge at ~ 372 nm. However, Ag@ZnO NCs with Ag NPs showed better absorption in the visible region from 450 to 550 nm, and the intensity of the absorption in the visible region is solely dependent on the absorption of Ag NPs^44^. The broad bands of NCs in the visible region are mainly due to the surface plasmon resonance (SPR) of Ag NPs indicating the polydispersity nature of nanoparticles. The increase in the plasmon peak intensity is correlated with the increase in the average size of the Ag NPs, and the absorption band for Ag NPs shifted towards a higher wavelength with the increasing Ag content. The bandgap energies (*Eg*) of all samples were calculated using the following equation: *αhν* = *A*(*hν–E*_*g*_)^*n*^*;* where *Eg* is the direct bandgap energy, *α* is the optical absorption coefficient, *hν* is the photon energy, *n* corresponds to the nature of transition, and *A* is the constant. The bandgap energies of all samples were calculated from Tauc’s plot, and the bandgap diagram and values are shown in Fig. 2b. The optical bandgap energy of ZnO was 3.1 eV, and the bandgap values of Ag@ZnO NCs decreased with increased Ag NPs binding on the surface of ZnO. The bandgap values were 2.99, 2.93, and 2.88 eV for Ag_0.2_@ZnO, Ag_0.4_@ZnO, and Ag_0.8_@ZnO NCs, respectively. The lower bandgap energies of Ag@ZnO NCs were attributed to the introduction of impurity into the ZnO grains that have trapped excited electrons from the conduction band and promoted a continuum of energy level and bandgap narrowing^45^. Besides, the GB extract components modify/stabilize the surface of NPs and NCs and reduce the bandgap values^46^.

The purity and crystal structure of ZnO and Ag@ZnO NCs, synthesized by the GB extract, were determined through powder XRD spectra, as shown in Fig. 2c. Powder XRD spectra revealed the characteristic 2θ peaks of ZnO at 31.76°, 34.43°, 36.26°, 47.56°, 56.61°, 62.90°, 66.41°, 67.98°, 69.10°, 72.61°, 77.0° corresponding for (100), (002), (101), (102), (110), (103), (200), (112), (201), (004), and (202) planes of the crystal lattices. This agrees with the polycrystalline diffraction patterns of the hexagonal phase of wurtzite ZnO (JCPDS card No. 89-0510), and no impurity peaks were observed^47^. Qu et al.^48^ demonstrated the synthesis of ZnO NPs with hexagonal wurtzite crystal structure from the *Sedum* *alfredii* Hance, a Zinc hyperaccumulating plant. In another instance, a bio-based approach was used to synthesize crystalline and polydispersed ZnO NPs (72.5 nm) using *Physalis* *alkekengi* L. to remediation of zinc-contaminated soils^49^. The average crystallite size of ZnO calculated using the Debye–Scherrer’s equation was 23.74 ± 4.9 nm. Also, the strong and sharp diffraction peaks confirm the high crystallinity of ZnO, and the degree of crystallinity was calculated through the equation: [area of crystalline peaks/area of crystalline & amorphous peaks] × 100, showed 100% crystallinity. Zaid et al*.*^50^ reported that calcination at higher temperatures could improve the crystallinity and better particle distribution. In Ag@ZnO NCs, the additional peaks of 38.11°, 44.30°, and 64.45° correspond to (111), (200), and (220) planes of face-centered cubic (fcc) phase of silver (JCPDS card No. 2-109)^51^. The ionic radius of silver ion (Ag^+^) (0.122 nm) was larger than that of zinc divalent (Zn^2+^) ions, thus silver ions cannot be substituted into the crystal lattice of the ZnO matrix; therefore, the metallic silver peaks due to the Ag NPs are formed over the ZnO surface^45^. The FWHM and crystallite size are inversely proportional; therefore, the increase in the size of Ag NPs results in the formation of larger NCs. These Ag NPs formed on the surface of ZnO were in the size of 25.65 ± 5.0, 32.91 ± 3.3, and 33.32 ± 4.21 nm in diameter for Ag_0.2_@ZnO, Ag_0.4_@ZnO, and Ag_0.8_@ZnO NCs, respectively. The intensity of Ag NPs peaks increases with the increase in the silver content of NCs, which is due to the increase in the number of Ag NPs on the surface of ZnO.

The functional groups involved in the formation of ZnO and Ag@ZnO NCs were investigated by the Fourier-transform infrared (FTIR) spectroscopy in the range of 400–4000 cm^–1^ (Fig. 2d). FTIR spectra of all samples and GB extract exhibited various absorption bands. In GB extract spectrum, the broad band centered at 3290 cm^–1^ was assigned to hydrogen-bonded O–H stretching vibrations and the weak signal at 2936 cm^–1^ was due to C–H stretching vibrations^52^. The band at 1595 cm^–1^ was attributed to the C–OH deformation vibration and the band at 1417 cm^–1^ was due to the O–C–O symmetric and asymmetric stretching vibrations of the carboxylate group. Moreover, the band at 1025 cm^–1^ was assigned to C–O stretching vibrations of the pyranose ring^30,53^. The FTIR spectra of ZnO and Ag@ZnO NCs exhibited a difference from the GB extract spectrum, the intensity of the broad band around wavenumber 3396 cm^–1^, the characteristic of OH stretching vibration, decreased in all samples after calcination^54^. Meanwhile, the broad absorption bands around 400–600 cm^–1^ were attributed to the stretching modes of metal–oxygen bonds, thus confirming the formation of Zn–O bonds^55^.

**Figure 3:**
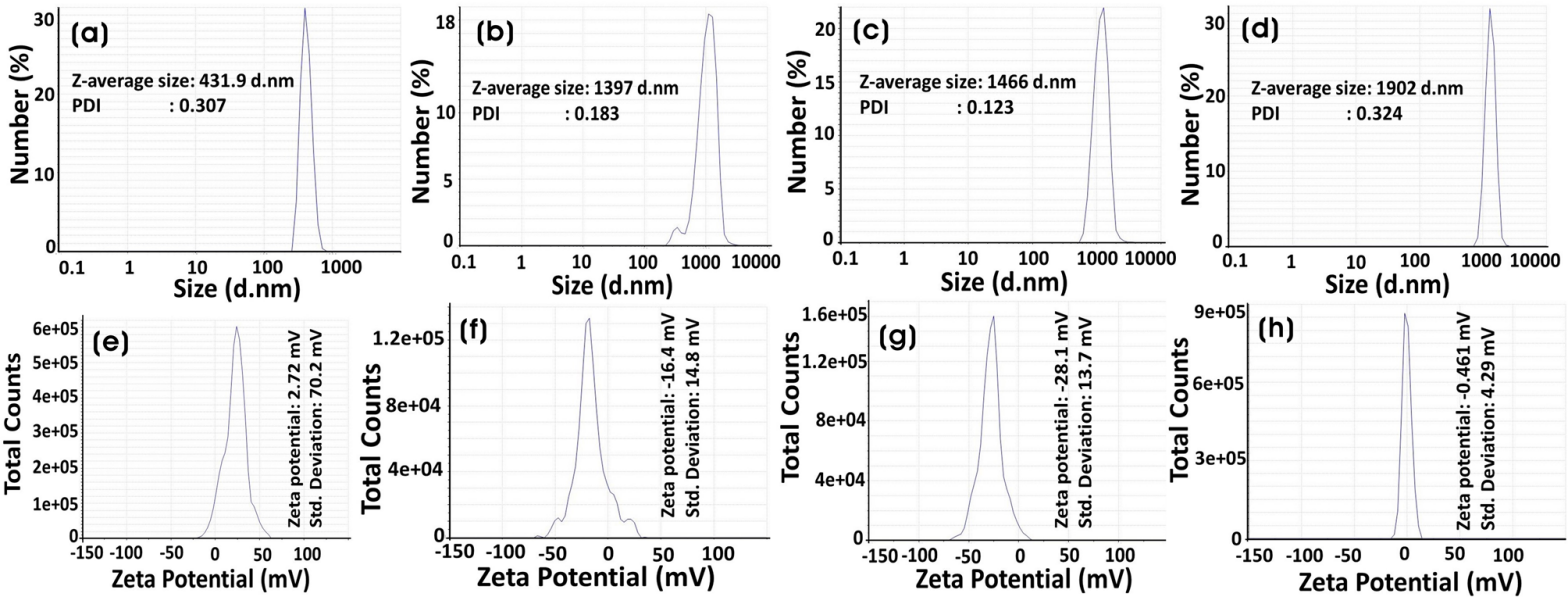
(**a**–**d**) Dynamic light scattering of particle size distribution and (**e**–**h**) zeta potentials of ZnO, Ag_0.2_@ZnO, Ag_0.4_@ZnO, and Ag_0.8_@ZnO NCs.

Dynamic light scattering (DLS) is a relatively robust and economical technique to measure the average size and size distribution of synthesized nanoparticles and nanocomposites. Mainly, DLS provides larger values because of the hydrodynamic shell, which is dependent on the structure, shape, and roughness of the particles^56^. According to Stokes–Einstein (SE) equation, the measured diffusion coefficients are related to the hydrodynamic radius as D = k_B_T/6πηR_h_, where k_B_ is Boltzmann’s constant (1.38 × 10^–23^ J/K), T is the temperature, η is the viscosity of the suspension medium, and R_h_ is the hydrodynamic radius^57^. There was an increase in the size of nanocomposites with the addition of silver to ZnO (Fig. 3a–d). The increase in the size can be caused by the formation of Ag NPs on the surface of ZnO particles and the aggregation of NCs. The zeta (ζ) potential is used to study the surface charges and stability of nano- or submicronic particles. The biomolecules from the GB extract were involved in reducing and stabilizing nanoparticles and nanocomposites. The zeta potential was calculated by dispersing the particles in water as the dispersion medium. The values of zeta potential were correlated with their stabilities: 0 to ± 5 (rapid coagulation), ± 10 to ± 30 (incipient stability), ± 30 to ± 40 (moderate stability), ± 40 to ± 60 (good stability), and >  ± 61 (excellent stability)^58,59^. The average zeta potential of synthesized ZnO after calcination was + 2.72 mV indicating positively charged groups in the stabilization. However, the zeta potentials of Ag_0.2_@ZnO, Ag_0.4_@ZnO, and Ag_0.8_@ZnO NCs were − 16.4, − 28.1, and − 0.46 mV, respectively (Fig. 3e–h). This shows that with the increase in the formation of Ag NPs on the surface of ZnO, the stability of Ag_0.2_@ZnO and Ag_0.4_@ZnO NCs increases, whereas the stability decreases with Ag_0.8_@ZnO NC.

**Figure 4:**
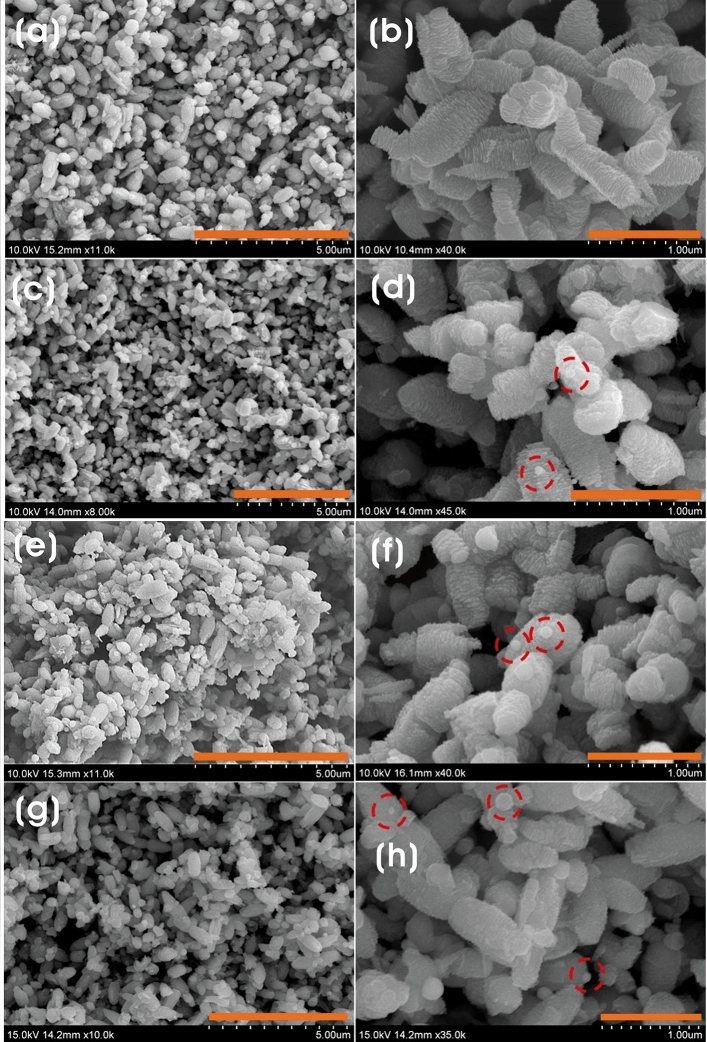
FE-SEM micrographs of (**a**,**b**) ZnO, and (**c**,**d**) Ag_0.2_@ZnO, (**e**,**f**) Ag_0.4_@ZnO, and (**g**,**h**) Ag_0.8_@ZnO NCs (scale bar 5 µm (left) and 1 µm (right)).

The surface morphology of ZnO and various Ag@ZnO NCs were identified using FE-SEM. Before calcination, the morphology of ZnO synthesized by precipitation method using GB extract as an additive promoted the formation of spike-like spherical morphology, which was different from the plate-like morphology of ZnO synthesized by direct precipitation method without any additive (Supplementary Fig. 1). This indicates the role of GB extract as a sustainable and eco-friendly material directing the formation of unique ZnO morphology. However, after the calcination process, the morphology of ZnO particles with GB extract was rearranged as clusters of ellipsoids with slight polydispersity on a submicronic scale. The ellipsoidal particles were in the size of 0.7 ± 0.13 and 0.38 ± 0.075 µm (length × width) (Fig. 4a,b). Remarkably, all ZnO particles were almost identical in dimension, and the surface looks puffy with an irregular pattern of pillar ridges. There was a slight agglomeration of particles due to the slightly higher surface area and durable affinity among ZnO particles^58^. Different morphologies of ZnO NPs, for example, nanospheres, nanoflower, nanoflakes, nanobelt, nanorods, nanowires, nanoneedles, nanotubes, and nanorings, can be synthesized by controlling the synthesis parameters^24,60,61^. The addition of silver with GB extract formed spherical Ag NPs i.e., 0.06 ± 0.011, 0.09 ± 0.04, 0.14 ± 0.045 µm for Ag_0.2_@ZnO, Ag_0.4_@ZnO, and Ag_0.8_@ZnO NCs, respectively, on the surface of ZnO particles. There was no significant change in the morphology of Ag@ZnO NCs except with the size of embedded Ag NPs on the surface of ZnO, which increased with the increase in the silver content added to the NCs (Fig. 4c–h).

**Figure 5:**
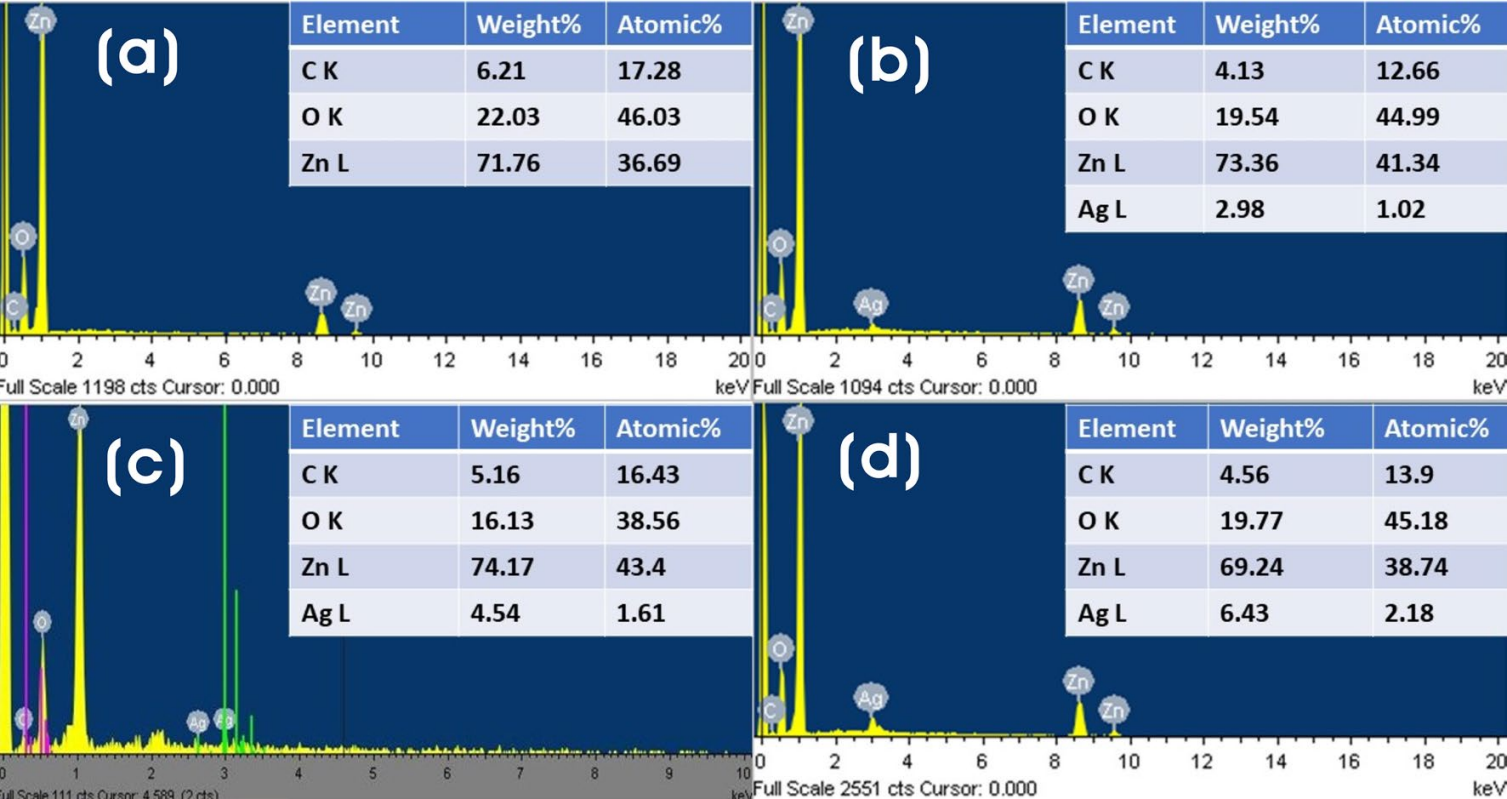
FE-SEM–EDX spectra of (**a**) ZnO, (**b**) Ag_0.2_@ZnO, (**c**) Ag_0.4_@ZnO, and (**d**) Ag_0.8_@ZnO NCs (inset table with elemental composition).

The elemental composition of ZnO and Ag@ZnO NCs was analyzed using the FE-SEM–EDX spectra, as shown in Fig. 5. The spectrum of ZnO particles showed a low energy peak at approximately 0.533 keV (O-K*α*) due to the presence of oxygen atoms, and other peaks for zinc and carbon atoms appeared at about 1.02 keV (Zn-L*α*), 8.6 keV (Zn-K*α*), 9.5 keV (Zn-K*β*), and 0.285 keV (C-K*α*). In contrast, Ag@ZnO NCs spectra contain intense low energy silver peaks at approximately 2.61 keV (Ag-K*α*), 3.0 keV (Ag-L*α*), 3.2 keV (Ag-L*β*), and 3.4 keV (Ag-L*β2*) along with Zn, O, and C peaks^62^. The EDX quantified silver, and other elements content in various Ag@ZnO NCs is shown in Fig. 5 (inset). The weight percent of silver increases from Ag_0.2_@ZnO to Ag_0.8_@ZnO, which infers the successful incorporation of silver as Ag NPs on ZnO^63^. Hence, the weight percentage of silver loaded on ZnO is proportional to the Ag concentration added to ZnO in the preparation of different Ag@ZnO NCs.

**Figure 6:**
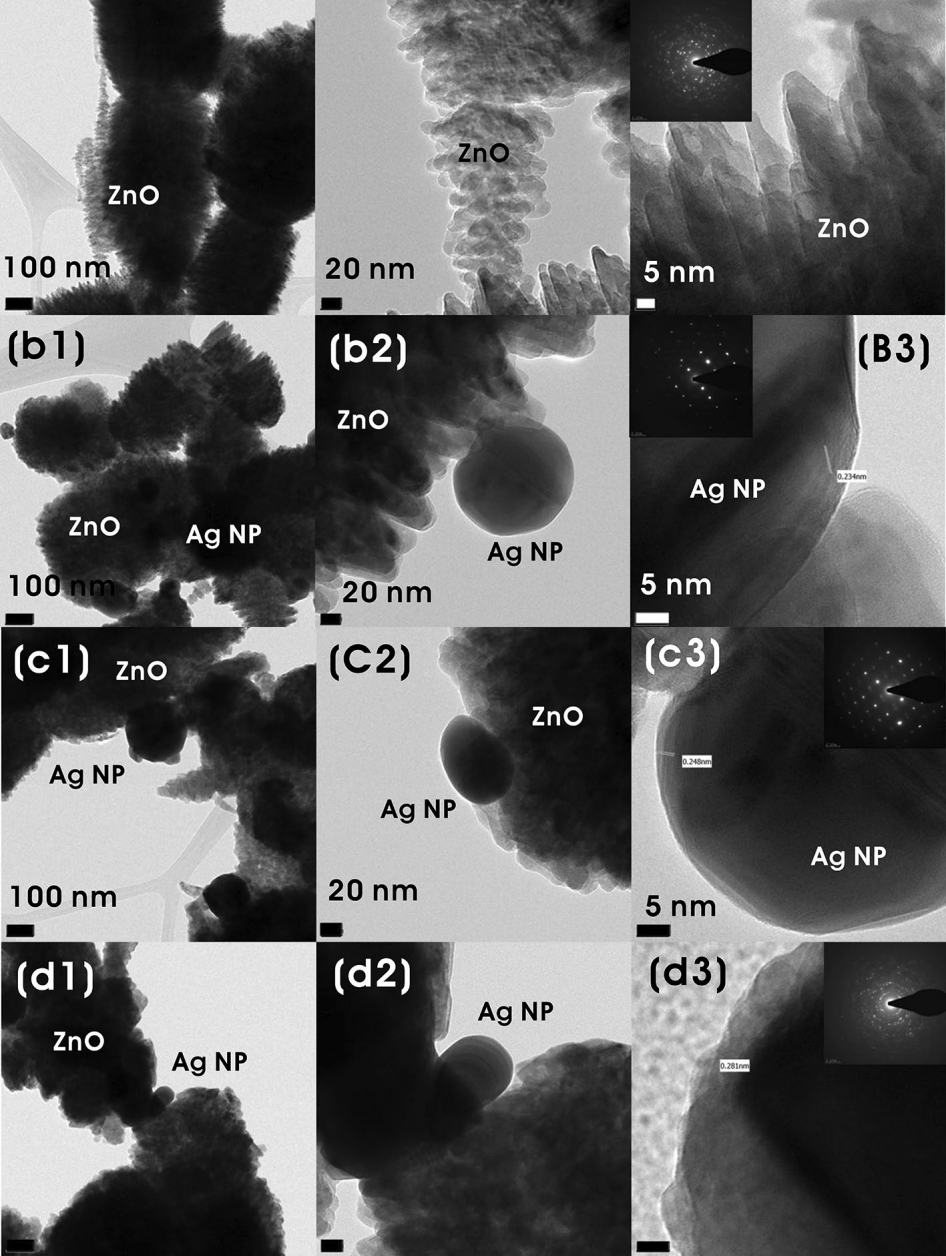
FE-TEM images of (**a1**–**a3**) ZnO, and (**b1**–**b3**) Ag_0.2_@ZnO, (**c1**–**c**3) Ag_0.4_@ZnO, and (**d1**–**d3**) Ag_0.8_@ZnO NCs, and the inset shows their corresponding SAED image.

FE-TEM analysis of ZnO and different Ag@ZnO NCs are shown in Fig. 6. Agglomeration of ellipsoidal ZnO submicronic particles and the formation of spherical Ag NPs on the ZnO surface were further verified by the FE-TEM results. It was found that ZnO was about 0.6 ± 0.11 and 0.33 ± 0.087 µm (length × width), whereas Ag_0.2_@ZnO, Ag_0.4_@ZnO, and Ag_0.8_@ZnO NCs have Ag NPs with the size of 87 ± 55, 130 ± 43 and 160 ± 55 nm, respectively. Furthermore, these results corresponded to the poor correlation between the FE-TEM sizes and particle size distribution analysis. With the increase in the silver content in the NCs, there was an occurrence of large particles due to the aggregation of small or primary particles. The ‘*d*’ spacing of ~ 0.281 nm between the adjacent lattice planes could be attributed to the (002) plane of ZnO (Fig. 6-d3). Similarly, the lattice fringes with *d* =  ~ 0.24 nm could be attributed to the (111) planes of Ag NPs (Fig. 6-b,c3). All these results confirmed the successful formation of Ag NPs on the surface of ZnO. The *d-*spacing of the (002) plane of ZnO in Ag@ZnO NC is like that of undoped ZnO, suggesting that Ag^+^ ions are not incorporated into the ZnO lattice. SAED pattern of ZnO clearly showed well-resolved diffraction rings indicating the polycrystallinity, and Ag NPs on ZnO (Ag@ZnO NCs) showed bright spots indicating the monocrystalline nature.

**Figure 7:**
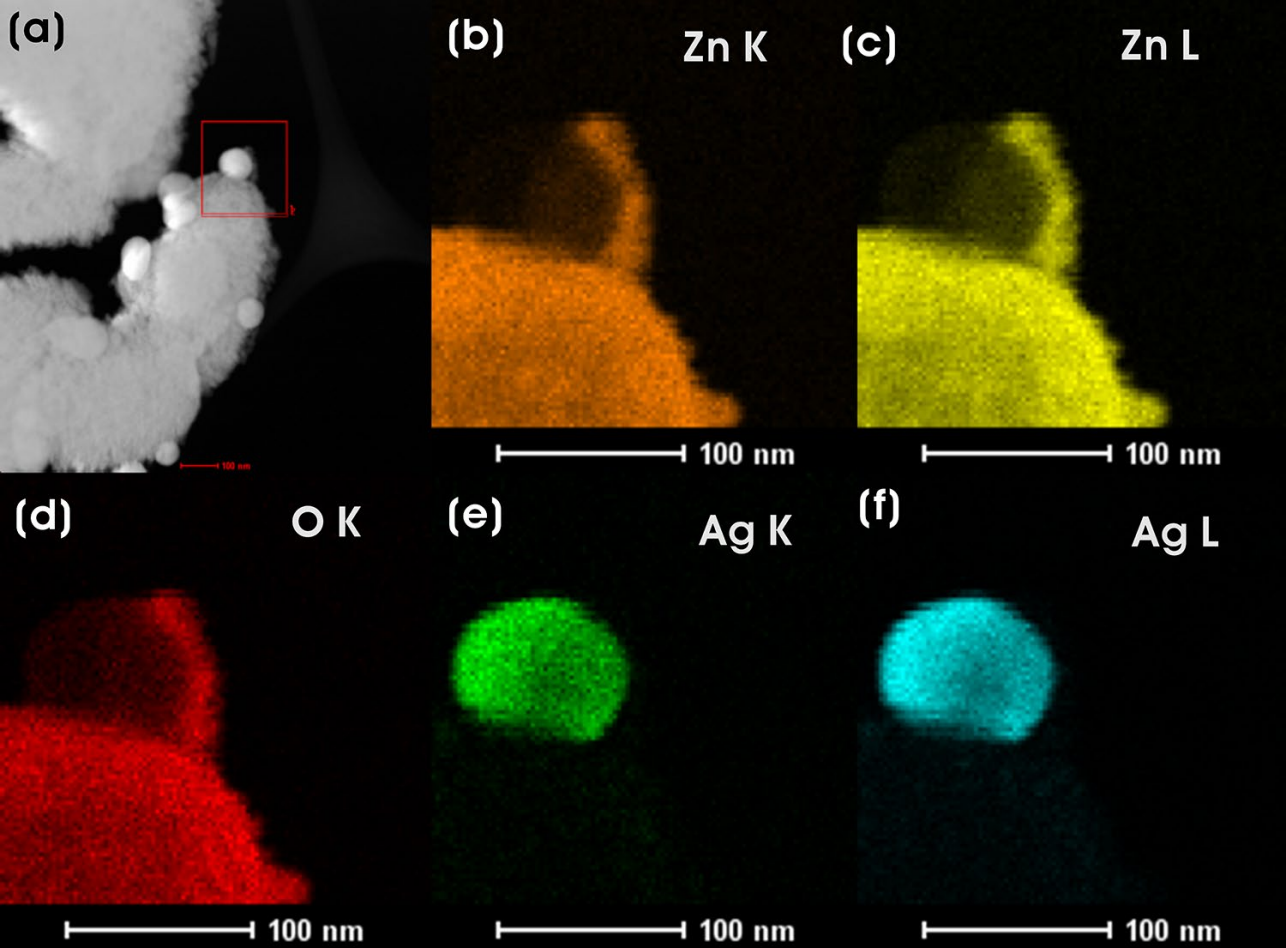
(**a**) HAADF-STEM image and EDX maps of (**b**) Zn-K, (**c**) Zn-L, (**d**) O-K, (**e**) Ag-K, and (**f**) Ag-L in Ag_0.2_@ZnO NC.

Figure 7 shows the HAADF-STEM image of the Ag_0.2_@ZnO NC and its corresponding elemental composition (Zn-K, Zn-L, O-K, Ag-K, and Ag-L) by STEM-EDX mapping. These images confirm the successful embedment of Ag NPs on the surface of ZnO.

**Figure 8:**
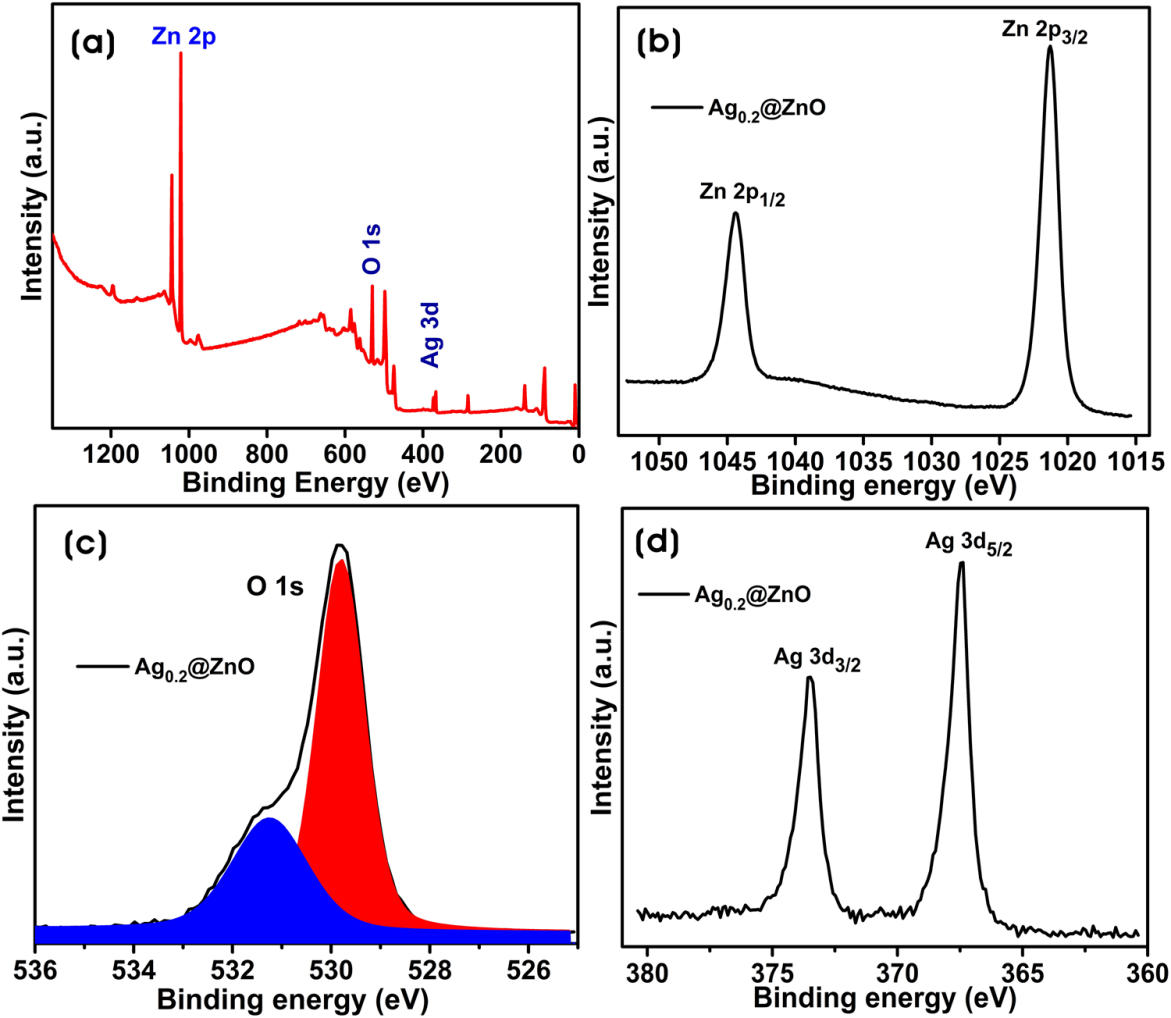
XPS spectrum of Ag_0.2_@ZnO NC. (**a**) Full survey spectrum, (**b**) Zn 2p spectrum, (**c**) deconvoluted O 1 s spectrum, and (**d**) Ag 3d spectrum.

XPS analysis was performed to clarify the chemical states of elements in Ag@ZnO NCs. The full scan survey of Ag_0.2_@ZnO NC shows the signals from Zn, O, and Ag elements with their corresponding atomic percent of 38.88, 37.34, and 1.63% in the range 0–1350 eV (Fig. 8a). Figure 8b shows the high-resolution spectra of Zn 2p. The peaks of Ag_0.2_@ZnO NC were located at 1021.28 eV and 1044.38 eV, which were ascribed to Zn 2p_3/2_ and Zn 2p_1/2_, respectively. These peaks confirm that the Zn element exists in a divalent cation (Zn^2+^) state in the NC. Figure 8c shows the high-resolution O 1 s peak of Ag_0.2_@ZnO NC. The deconvoluted O 1 s peak shows two subpeaks at binding energies of 529.8 and 531.2 eV attributing to the lattice oxygen of ZnO and dissociated oxygen or hydroxyl-like group on the surface of ZnO, respectively^64,65^. The presence of surface hydroxyl groups acts as adsorption sites of dyes and reacts with photogenerated holes forming hydroxyl radicals by oxidation, which decomposes dyes during photodegradation^66^. Therefore, the presence of a surface hydroxyl group with 28.9% was one of the critical factors in the photodegradation process. Figure 8d shows the high-resolution spectrum of Ag 3d photoelectron peaks of Ag_0.2_@ZnO NC. The Ag 3d spectrum shows two peaks centered at 367.38 and 373.48 eV ascribed to Ag 3d_5/2_ and Ag 3d_3/2_ transitions, respectively. The difference in the binding energy of ~ 6.0 eV between Ag 3d_5/2_ and Ag 3d_3/2_ peaks was the characteristic of metallic silver and consistent with the results of XRD analysis^30,67^.

**Figure 9:**
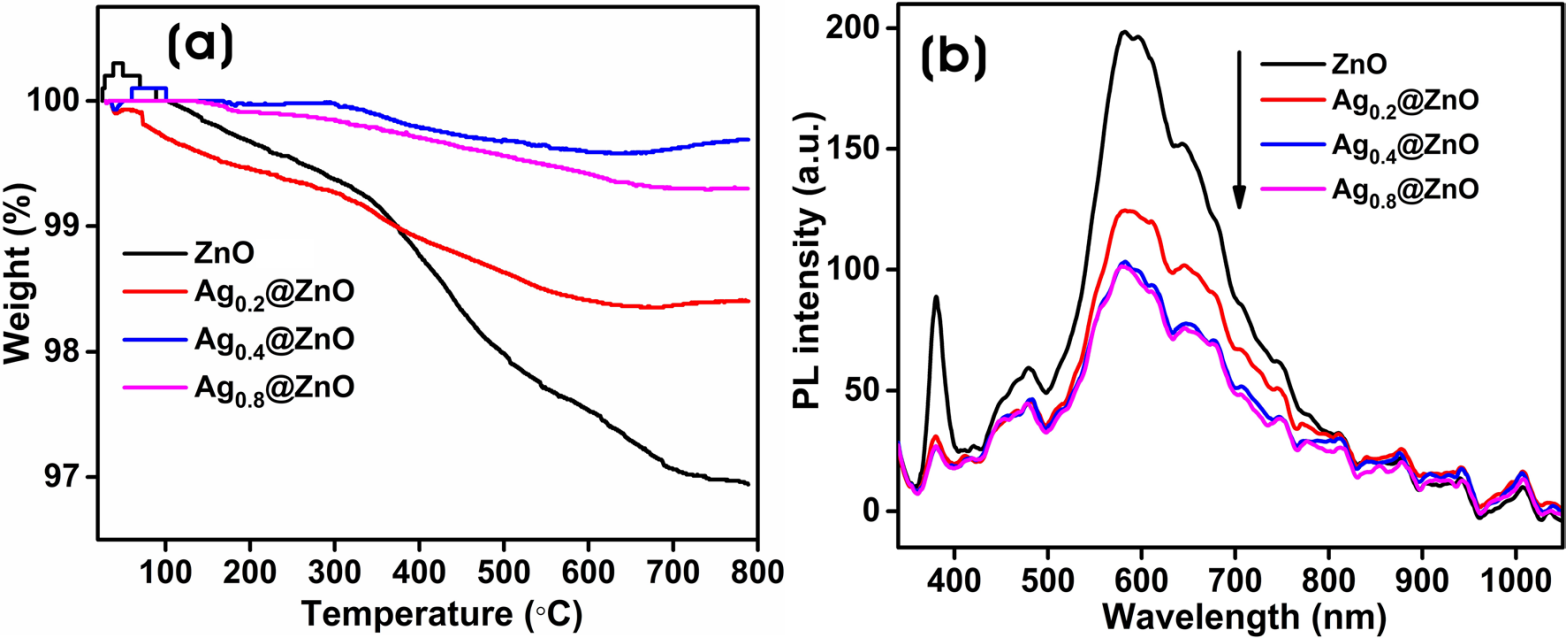
(**a**) TG analysis curve and (**b**) PL spectra of ZnO and Ag@ZnO NCs.

Figure 9a shows the thermogravimetric (TG) analysis of ZnO, Ag_0.2_@ZnO, Ag_0.4_@ZnO, and Ag_0.8_@ZnO NCs. TG analysis was performed from room temperature to 800 °C at a rate of 20 °C/min in a nitrogen atmosphere to demonstrate the thermal stabilities of ZnO and Ag@ZnO NCs. The overall weight loss for all samples was very minimal. A total weight loss of 3.0, 1.6, 0.3, and 0.7% occurred for ZnO, Ag_0.2_@ZnO, Ag_0.4_@ZnO, and Ag_0.8_@ZnO NCs, respectively, and all NCs are highly thermally stable. In ZnO, at the low-temperature range (up to 90 °C), the weight seems to have increased due to the OH bonding with the reaction with moisture. Further, the weight loss up to 350 °C, accounting for ~ 0.83% was attributed to the loss of H_2_O molecules and evaporation of gases that were physically and chemically adsorbed on the surface of ZnO particles^68^. This explains that ZnO absorbs nitrogen and slowly releases them over a period, which indicates that ZnO was pure and very porous in nature^69^. The weight loss accounting for ~ 2.1% from 350 to 700 °C was higher with ZnO; this could be due to the thermal decomposition of biomolecules of GB extracts, such as phenolic compounds and other metabolites. Above 700 °C, there was no significant weight loss in ZnO. A similar decomposition pattern was observed with Ag_0.2_@ZnO NC; however, the embedment of silver slightly improved the thermal stability and decomposition of Ag_0.2_@ZnO NC compared to that of ZnO. In Ag_0.4_@ZnO and Ag_0.8_@ZnO NCs, the continuous decrease in weight in the nitrogen environment was attributed probably to the oxygen out-diffusion from the ZnO matrix resulting in the formation of oxygen-deficient ZnO compound (ZnO_1-δ_).

PL spectrum is a valuable tool to investigate the state of photogenerated e‾/h^+^ pairs and the defects of metal/semiconductor nanocomposites^70^. Figure 9b shows the PL spectra of ZnO and Ag@ZnO NCs at room temperature. There are two distinct emission peaks in the UV region (~ 380 nm) and visible region (~ 400 to 750 nm). These emission peaks provide information about the recombination between charge carriers and defect levels^21^. The emission peak at ~ 380 nm in ZnO corresponds to near band edge emission (NBE), attributed to bandgap excitation^71^. The broad band emission extending from ~ 400 to 750 nm in the whole visible spectrum can be from deep-level emission (DLE), i.e., because of crystal defects like Zn-interstitials and oxygen vacancies^55,72^. All Ag@ZnO NCs showed decreased PL intensity than ZnO, which suggests the decrease in the recombination rate of photoinduced electrons and holes with the embedment of Ag NPs favoring the photocatalytic activity than ZnO^73^. The PL intensity of Ag_0.2_@ZnO NCs decreased drastically with the increase in the silver content in the NCs providing the separation of photoinduced e‾/h^+^ pairs and inhibiting the recombination of photoinduced pairs^74,75^. However, at Ag_0.8_@ZnO NC, with the increase in the Ag concentration, there was no increase in the PL intensity and overlapped with the peaks of Ag_0.4_@ZnO NC, suggesting the formation of new recombination centers, which are unfavorable to the separation of photoinduced pairs^70,76^. Thus, Ag_0.8_@ZnO NCs exhibited the lowest PL intensity as that of Ag_0.4_@ZnO NC because excess addition of silver as Ag NPs in Ag_0.8_@ZnO NC was unfavorable for charge separation.

**Figure 10:**
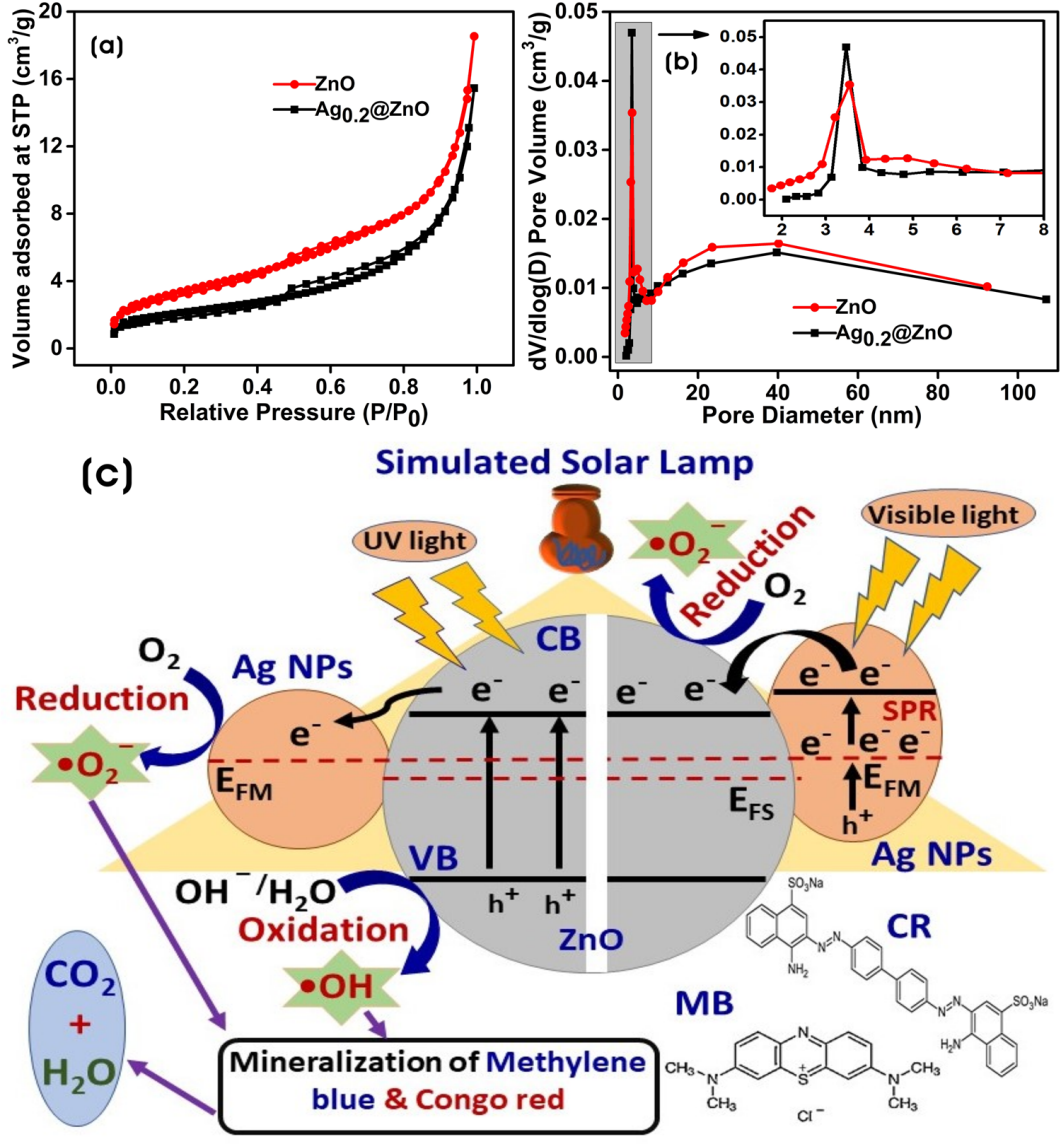
The N_2_ adsorption–desorption isotherm of ZnO and Ag_0.2_@ZnO NC. (**a**) BET surface area analysis (*S*_*BET*_), and (**b**) pore size distribution and (**c**) schematic diagram illustrating the photocatalytic degradation of MB and CR dyes by Ag@ZnO NCs.

To determine the structural and adsorption parameters of ZnO and Ag_0.2_@ZnO NC, nitrogen (N_2_) adsorption–desorption isotherms at 77 K were recorded. Figure 10a shows the N_2_ adsorption–desorption isotherms of ZnO and Ag_0.2_@ZnO NC. According to IUPAC classification, these curves obtained for evaluating surface area were approximately identical to that of Type IV isotherm with H_3_ hysteresis loop^77^. The well-defined inflection around relative pressure (P/P_0_) of 0.5–0.9 indicates the presence of a heterogeneously distributed mesoporous nature of particles^78^. The BET surface area (*S*_*BET*_) was determined from isotherms using the BET equation^79^. The values of *S*_*BET*_ were found to be 11.77 and 7.5 m^2^/g for ZnO and Ag_0.2_@ZnO NC, respectively, and the mesoporous material contains narrow pores that hinder the movement of nitrogen and limits the adsorption. The *S*_*BET*_ of Ag_0.2_@ZnO NC decreased with the embedment of Ag NPs more than ZnO, revealing that the formed Ag NPs could have occupied and blocked the pores of ZnO. Figure 10b shows the pore size distribution curve obtained using the Barrett–Joyner–Halenda (BJH) model. It could be seen that most of the pores were in the size range of 2–40 nm, which provides evidence for the mesoporosity framework of ZnO and Ag_0.2_@ZnO NC. The BHJ average pore sizes of ZnO and Ag_0.2_@ZnO NC were 9.52 and 11.57 nm, respectively, and the calculated mean pore volumes were 0.027 and 0.023 cm^3^/g. The *S*_*BET*_, BHJ mean pore size, and pore volume of Ag_0.2_@ZnO NC were lower than that of ZnO because of the embedment of Ag NPs on the surface of ZnO.

### Mechanism of photocatalytic activity

The schematic diagram of the photocatalytic degradation of dyes MB and CR by ZnO and Ag@ZnO NCs is proposed in Fig. 10c. The advanced oxidation processes (AOPs) generate ROS of highly reactive species such as superoxide anion radicals (^·^O_2_‾), and hydroxyl radicals (^·^OH) are mainly involved in the degradation and mineralization of dyes into carbon dioxide (CO_2_) and water^80^.

When ZnO is irradiated by the UV light of the simulated solar lamp, electrons in the valence band (VB) get excited to the conduction band (CB), leaving behind holes in the VB^81^. These photogenerated electrons get transferred to the Ag NPs as the CB energy level of ZnO is higher than the Fermi level (E_FM_) of metallic Ag, which hinders the recombination and extends the lifetime of photogenerated (e‾/h^+^) pairs, whereas Ag NPs in the NCs absorb visible light undergo surface plasmon resonance (SPR), and these excited electrons in the 3d orbit of Ag NPs get easily transferred to CB of ZnO owing to the interface effect of Ag/ZnO heterojunctions, yielding more superoxide anion radicals. The holes formed by the excitation of electrons will generate ^·^OH radicals by oxidation of hydroxyl ions. Thus, the as-formed superoxide anion radicals and hydroxyl radicals are mainly responsible for the effective mineralization of dyes into CO_2_ and water^82,83^. The increase of silver amount on the surface of ZnO decreases the photocatalytic degradation efficiency. The decrease in the photocatalytic degradation by Ag_0.4_@ZnO and Ag_0.8_@ZnO NCs could be due to the hindrance in the absorption of light by the excess of Ag NPs, which is in agreement with the PL results.

## Applications of ZnO and Ag@ZnO NCs

### Photocatalytic degradation of dyes

**Figure 11:**
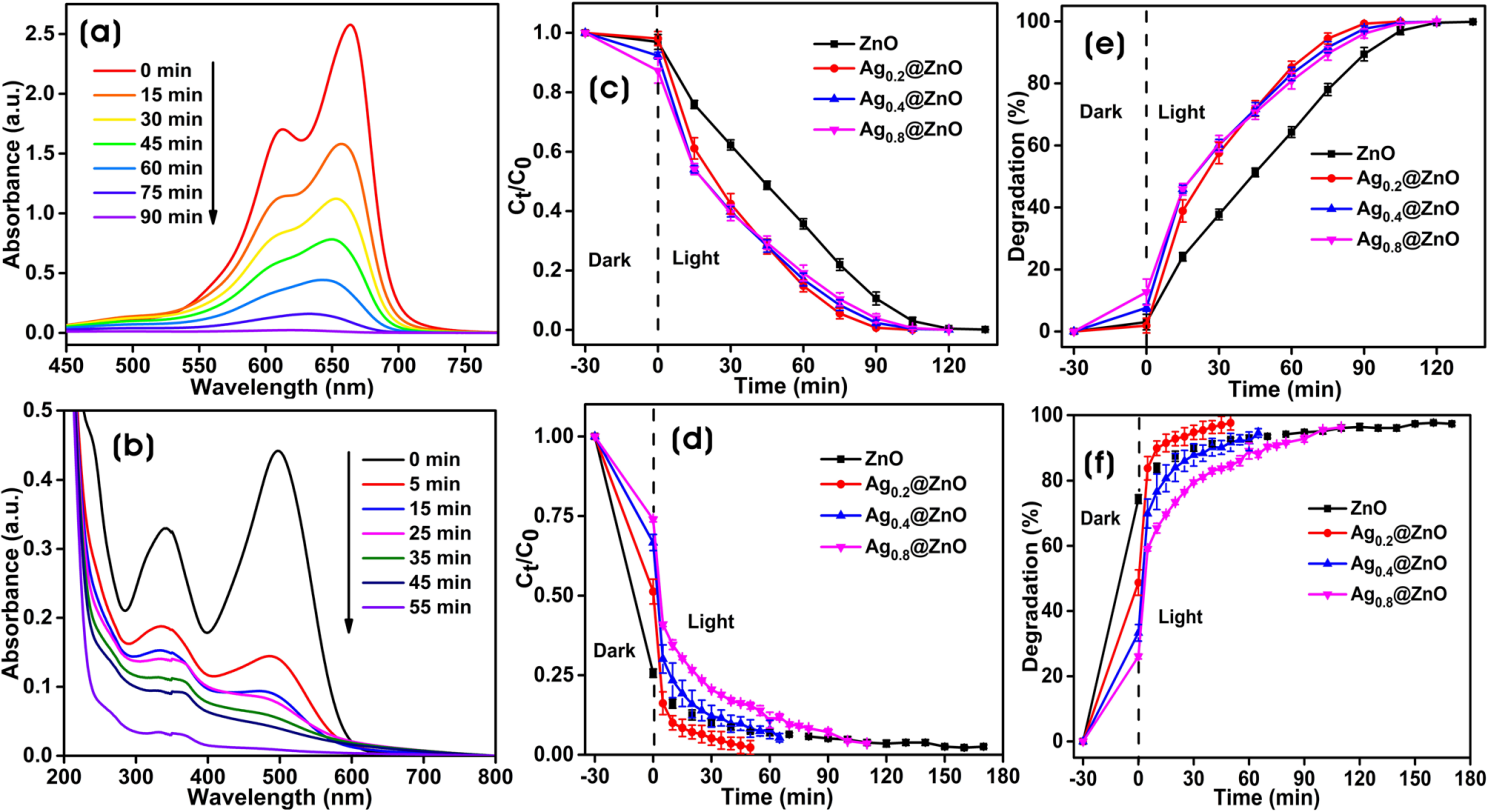
UV–Vis absorbance spectra of the photocatalytic degradation of MB (**a**) and CR (**b**). The plot of C_t_/C_0_ against time in the degradation of MB (**c**) and CR (**d**). The degradation percentage of MB (**e**) and CR (**f**) against time by Ag_0.2_@ZnO NC.

Photocatalysis happens on the surface of the photocatalyst, and the photocatalytic performance of ZnO was ameliorated by increasing the surface-to-volume ratio and by modifying the band structure by the incorporation of Ag NPs to improve the visible-light absorption properties and thereby efficiently restricting the recombination of photogenerated (e‾/h^+^) pairs^21,84,85^. The photocatalytic properties of ZnO and Ag@ZnO NCs were evaluated via the degradation of dyes MB (cationic) and CR (anionic) under the simulated solar lamp. Figure 11a shows the UV–Vis absorption spectra of the degradation of MB with time in the presence of ZnO and Ag@ZnO NCs. Figure 11c shows the photocatalytic degradation (C_t_/C_0_) as a function of time, where C_t_ is the concentration of MB at the time “t”, and C_0_ is the initial concentration. The experimental solution containing the MB (1.0 mg/100 mL) and photocatalyst (0.1% w/v) was allowed for the adsorption–desorption equilibrium in the dark for 30 min, and the MB dye in the range of 3.0 ± 2.5–12.7 ± 4.2% was adsorbed on ZnO and Ag@ZnO NCs. The increase of silver content as Ag NPs on ZnO increased the adsorption of MB dye on its surface. Moreover, the strong MB dye adsorption capacities by Ag@NCs in the dark improved their photocatalytic performances in terms of their decolorization and degradation processes^86^. The degradation percentage of MB by ZnO, Ag_0.2_@ZnO, Ag_0.4_@ZnO, and Ag_0.8_@ZnO NCs were 89.4 ± 2.2, 99.2 ± 0.34, 97.6 ± 0.91, and 96.0 ± 0.1.5%, respectively after irradiation for 90 min (Fig. 11e). Ag_0.2_@ZnO as photocatalysts showed higher photocatalytic degradative activity than other NCs and ZnO, and it showed 100% degradation in 90 min. However, other Ag@ZnO NCs and ZnO showed ~ 100% photocatalytic degradation in 120 min. The higher photocatalytic activity by Ag_0.2_@ZnO NC suggests that adding silver to ZnO improves the photocatalytic activity significantly.

Similarly, the UV–Vis absorption spectra of the degradation of CR dye with time by ZnO and different Ag@ZnO NC were shown in Fig. 11b. The C_t_/C_0_ degradation of CR versus time is shown in Fig. 11d. After incubation at dark for attaining adsorption–desorption equilibrium, CR dye of 26.0 ± 0.77–74.4 ± 1.3% was adsorbed onto ZnO and Ag@ZnO NCs. ZnO had a strong adsorption ability of CR on its surface; however, with an increase in the silver amount, the adsorption of CR on the surface of NCs decreases. Moreover, the strong adsorption capacities of ZnO and Ag@ZnO NCs in the dark improved their photocatalytic performances in their decolorization and degradation processes. The degradation percentages of CR by ZnO, Ag_0.2_@ZnO, Ag_0.4_@ZnO, and Ag_0.8_@ZnO NCs were 92.9 ± 0.5, 98.4 ± 2.4, 92.5 ± 1.5, and 86.1 ± 1.5%, respectively after irradiation for 55 min (Fig. 11f; Table 1). Analogous to MB degradation, the degradation of CR by Ag_0.2_@ZnO NC was higher than other Ag@ZnO NCs and ZnO. The photolysis of dyes without photocatalyst was also determined. Both dyes are barely degraded without photocatalyst, which indicates that both MB and CR dyes are stable in the aqueous environment under simulated solar irradiation. However, CR appears to be more stable than MB under the experimental conditions. There was photolysis of 26.97% and 4.26% for MB and CR dyes, respectively, after irradiation for 150 and 210 min (Supplementary Figs. 2, 3). Among different metal/semiconductor NCs prepared using plant/fruit extracts, Ag@ZnO NCs prepared using GB extract as an additive yielded unique ellipsoidal morphology with mesoporosity, and the photodegradation efficiency of MB and CR was comparable to the previous reports (Table 1). Reusability of the catalyst is one of the important intentions for photocatalytic reactions. The use of powdered catalysts has been limited due to the difficulties involved in the separation of catalysts after the degradation of pollutants. But, nowadays, present catalysts can be easily separable from the solution either by filtration or by centrifugation, and there is no permanent adsorption of dyes over the photocatalyst^87^. Regeneration of photocatalyst after every reaction was done by collecting the catalyst by centrifugation, washing with water, and calcination at 200 °C for 1 h. The photocatalytic activity of Ag_0.2_@ZnO NC remains intact for both MB and CR degradation for up to five adsorption/desorption cycles under selected conditions. There was only an indiscernible decrease of about 8.1% in the photocatalytic degradation of MB. However, there was a significant decrease of about 33% in the degradation of CR (Supplementary Fig. 4). Thus, the recycling results reflect the commendable stability of both Ag NPs and ZnO structures in the Ag_0.2_@ZnO NC for the degradation of cationic dyes for wastewater treatment.

**Table 1:** List of Ag/ZnO NCs synthesized using plant/fruit extracts and their photocatalytic degradation of dyes and antibacterial activities.

Plant/fruit extract	Irradiation	[Dye]	[Ag-ZnO]		Degradation (%)	Degradation time (min)	Pathogens	References
*Ocimum* *tenuiflorum* (Seed extract)	Solar	MB (10 ppm)	0.5%		74.41	120		
			1.0%		94.27	120		
			2.0%		49.2	120	*E.* *coli*	^51^
*Solanum* *tuberosum* (Potato peels)	Visible (250 W)	MB (5 ppm)	0.01%	(1.0 wt% Ag)	72.0	80		
				(2.0 wt% Ag)	~96	80		
				(8.0 wt% Ag)	85.0	80		
				(10.0 wt% Ag)	89.0	80	NA	^84^
*Psidium* *guajava* (Leaf extract)	Solar (800 W/m^2 ^	MB (22.4 ppm)	0.04%	(2.5 mol% Ag)	98%	60	NA	^85^
*Thymus* *vulgaris* (Leaf extract)	Solar	Phenol (20 mg/L)	60 ppm		97.2	120	*E.* *coli* & *S.* *aureus*	^93^
*Trigonella* *foenum-graecum* (Leaf extract)	Visible* (400 W)	MG (20 ppm)	0.1%		100	120	*E.* *coli* & *S.* *aureus*	
							*C.* *albicans*	^92^
*Lycium* *barbarum* L. (Fruit extract as an additive)	Solar^#^ (300 W)	MB (10 ppm)	0.1%		99.3	90		
		CR (20 ppm)	0.1%		98.5	55	*E.* *coli* & *S.* *aureus*	(This study)

NA: not applicable, MB: methylene blue, MG: malachite green; *Hg lamp (λ > 420 nm); ^#^simulated solar lamp.

### Antibacterial assay

**Figure 12:**
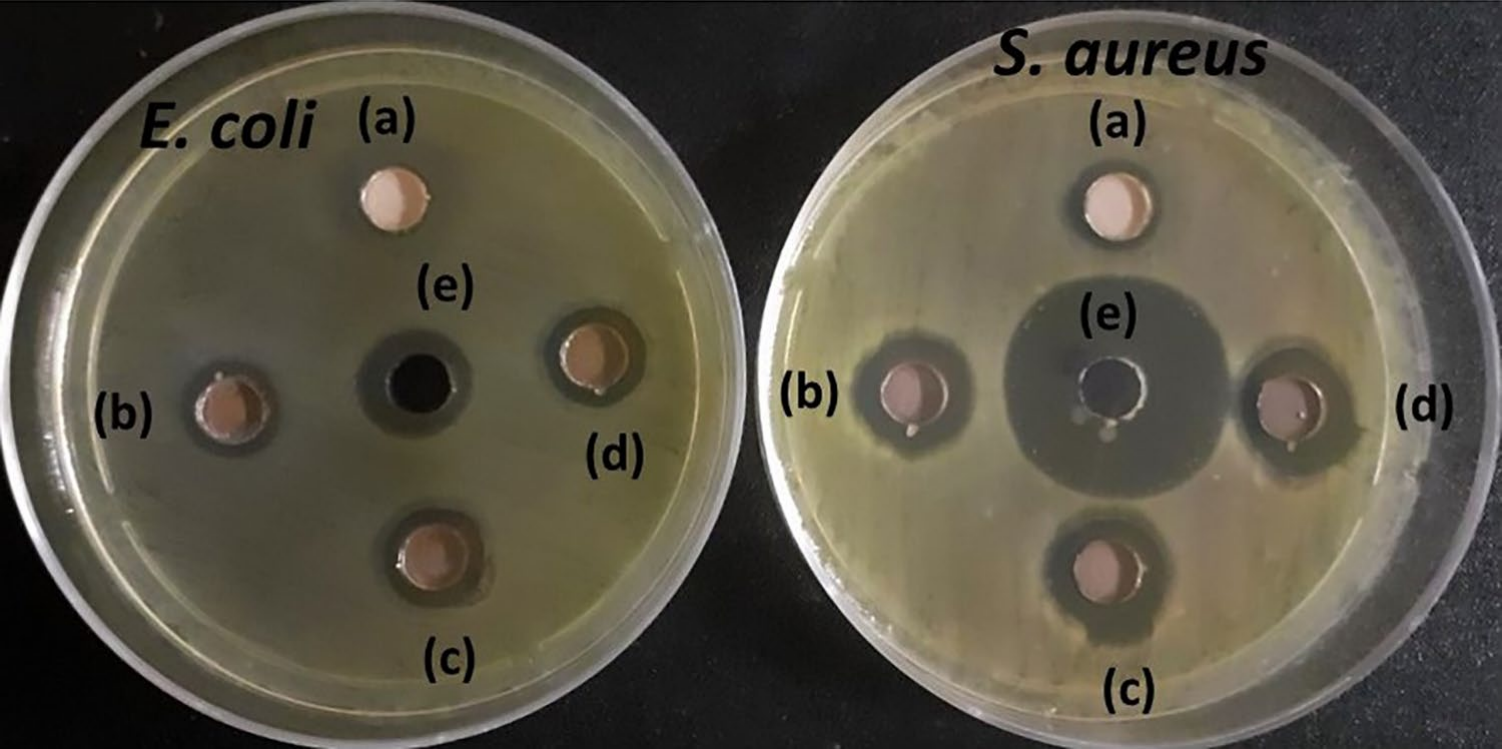
Antibacterial activity showing ZOIs for (**a**) ZnO, (**b**) Ag_0.2_@ZnO, (**c**) Ag_0.4_@ZnO, (**d**) Ag_0.8_@ZnO NCs (2 mg; 40 mg/mL), and (**e**) ampicillin (positive control) against *E.* *coli* and *S.* *aureus*.

Figure 12 shows the antibacterial activity of ZnO and other Ag@ZnO NCs against Gram-positive (*S.* *aureus*) and Gram-negative (*E.* *coli*) bacteria. The antibacterial activity was evaluated using the agar well diffusion method, which shows that both *E.* *coli* and *S.* *aureus* were susceptible to all Ag@ZnO NCs. However, *E.* *coli* showed slight resistance to the antimicrobial activity by ZnO and Ag@ZnO NCs. The zones of inhibitions (ZOIs) for Ag_0.2_@ZnO, Ag_0.4_@ZnO, and Ag_0.8_@ZnO NCs were 11.0 ± 0.4, 11.4 ± 0.5, and 11.3 ± 0.6 mm, respectively, for *E.* *coli*, whereas it was 13.8 ± 0.6, 14.4 ± 1.0, and 14.6 ± 0.9 mm for *S.* *aureus*. Moreover, ZnO showed a marginal ZOI of 10.2 ± 0.4 mm only in *S.* *aureus*. The ZOIs for the positive control (ampicillin) were 12.6 ± 0.5 and 25.6 ± 0.7 mm for *E.* *coli* and *S.* *aureus*, respectively. Our previous study found that GB extract does not possess antibacterial activity against *E.* *coli* and *S.* *aureus*^30^. Thus, all Ag@ZnO NCs at a 2 mg (0.05 mL) concentration exhibited broad-spectrum antibacterial activity against both *E.* *coli* and *S.* *aureus*. The antibacterial activity of the ZnO is most likely due to the release of ROS on the surface of ZnO, which disrupts the bacterial membrane; in particular, the production of hydrogen peroxide (H_2_O_2_) penetrates the cell membrane and kills the microorganism^88,89^. Gunalan et al*.*^90^ demonstrated the antibacterial activity of *Aloe* leaf extract-mediated synthesis of nano-ZnO against *S.* *aureus*, *Serratia* *marcescens*, *Proteus* *mirabilis*, and *Citrobacter* *freundii*. Even Ag NPs in Ag@ZnO NCs can cause membrane permeation and bacterial ROS production for the synergistic antibacterial activity with ZnO particles in the nanocomposite^91,92^. Zare et al*.* ^93^ evaluated the antibacterial potency of ZnO-Ag NC on bacteria. They proposed that physical interaction with bacterial cells causes disruption of cell membrane and oxidization of cell components for exhibiting broad-spectrum antibacterial activity against multidrug-resistant bacteria.

## Conclusions

The synthesis of zinc oxide particles (ZnO) by direct precipitation method using goji berry extract as an additive and subsequent calcination in air promoted the formation of mesoporous ellipsoidal morphology with 0.59 µm (length), and 0.33 µm (width) was found to be hexagonal wurtzite crystal structure. The formation of silver nanoparticles on the surface of ZnO in the formation of Ag@ZnO nanocomposites using the GB extract provides a method of synthesizing highly porous metal/semiconductor NCs. The presence of polyphenols in the GB extract acts as both reducing and capping/stabilizing agents in preparing nanoparticles and/or nanocomposites. The as-prepared Ag@ZnO NCs were characterized by several techniques, such as FT-IR, XRD, FE-SEM, TEM, EDS, XPS, and UV–Vis spectroscopy. The XRD analysis, and SEM–EDX and TEM micrographs confirmed the formation of Ag NPs on the surface of ZnO. The photocatalytic activity of Ag_0.2_@ZnO nanocomposite towards both MB and CR degradation in an aqueous medium was found to be higher than that of ZnO and other Ag@ZnO NCs at room temperature. Ag_0.2_@ZnO NC was photostable and reusable for cationic dyes even after five adsorption/desorption cycles. The presence of Ag on the surface of ZnO promotes the separation of photogenerated charge carriers and enhances photocatalytic degradation of pollutants. In addition, they also showed good antibacterial activity against *Staphylococcus* *aureus* and *Escherichia* *coli*. Both, the photocatalytic and antibacterial activity of Ag_0.2_@ZnO were remarkably improved due to the generation of abundant ROS than that of ZnO particles and other Ag@ZnO NCs. This novel methodology utilizes fruit extract as a sustainable and eco-friendly additive to form the unique morphology of semiconductor particles, and as a reducing/stabilizing agent to form metal nanoparticles to prepare metal/semiconductor nanocomposites for wastewater treatment by photocatalysis and antimicrobial therapeutics.

## Supplementary Information

Supplementary Information.

## Data Availability

Data available on request from the authors.
