# Supplementary material for: Photocatalytic degradation activity of goji berry extract synthesized silver-loaded mesoporous zinc oxide (Ag@ZnO) nanocomposites under simulated solar light irradiation
Source: Sci Rep. 2022 Jun 15;12:10017. doi: 10.1038/s41598-022-14117-w (PMC9200859; doi:10.1038/s41598-022-14117-w)
Supplement: Supplementary file 1 — Supplementary Information. [file 41598_2022_14117_MOESM1_ESM.docx]

**Photocatalytic degradation activity of goji berry extract synthesized silver-loaded mesoporous zinc oxide (Ag@ZnO) nanocomposites under simulated solar light irradiation**

Abdulrahman Ahmed Sharwani^a^, Kannan Badri Narayanan^a,b*^, Mohammad Ehtisham Khan^c^, Sung Soo Han^a,b*^

^a^School of Chemical Engineering, Yeungnam University, 280 Daehak-Ro, Gyeongsan, Gyeongbuk 38541, South Korea

^b^Research Institute of Cell Culture, Yeungnam University, 280 Daehak-Ro, Gyeongsan, Gyeongbuk 38541, South Korea

^c^Department of Chemical Engineering Technology, College of Applied Industrial Technology (CAIT), Jazan University, Jazan 45971, Saudi Arabia

*Corresponding authors

Dr. Kannan Badri Narayanan, Email ID: [okbadri@gmail.com](mailto:okbadri@gmail.com), [okbadri@yu.ac.kr](mailto:okbadri@yu.ac.kr)

Prof. Sung Soo Han, Email ID: [sshan@yu.ac.kr](mailto:sshan@yu.ac.kr)

Tel: +82–53–810–2773; Fax: +82–53–810–4686


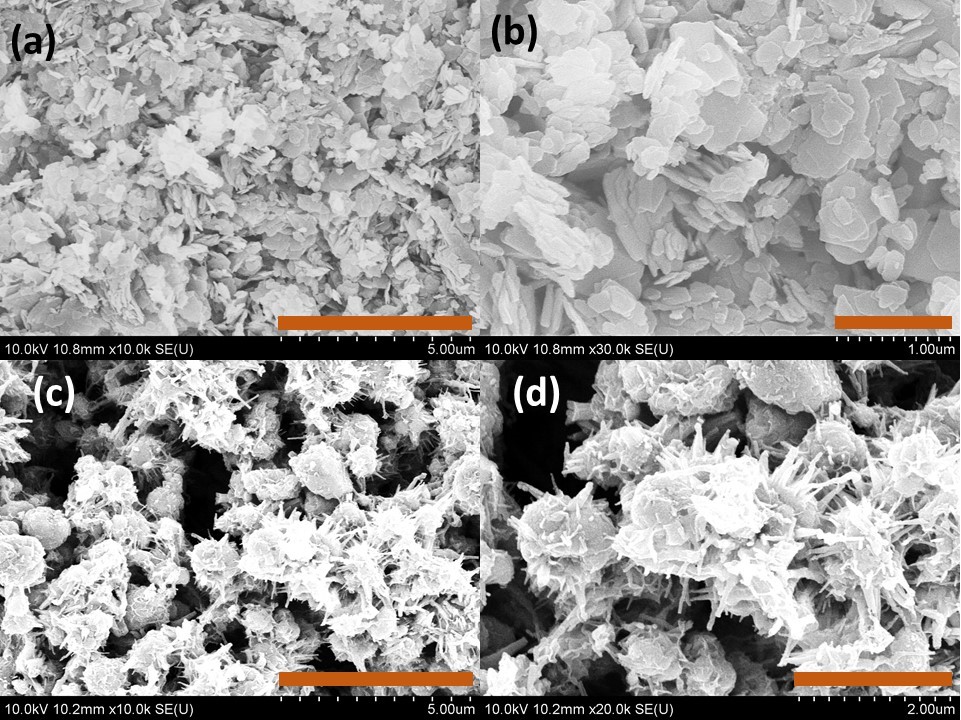


**Supplementary Figure 1.** ZnO synthesized by (a, b) precipitation method and (c, d) using GB extract as additive to precipitation method and dried at 60 °C (before calcination).


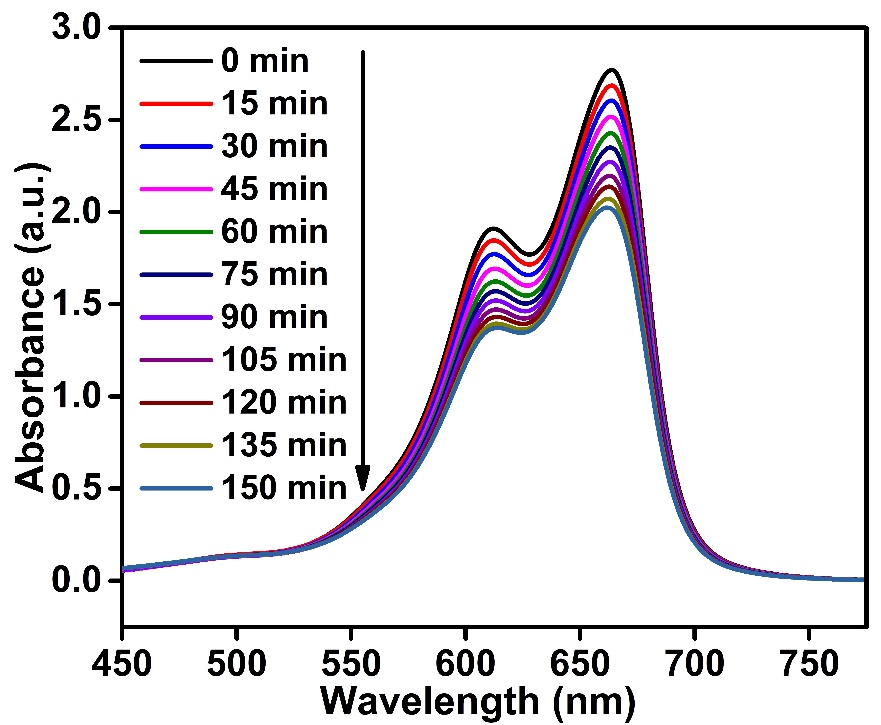


**Supplementary Figure 2.** Photolysis of methylene blue dye without Ag@ZnO NCs (photocatalyst).


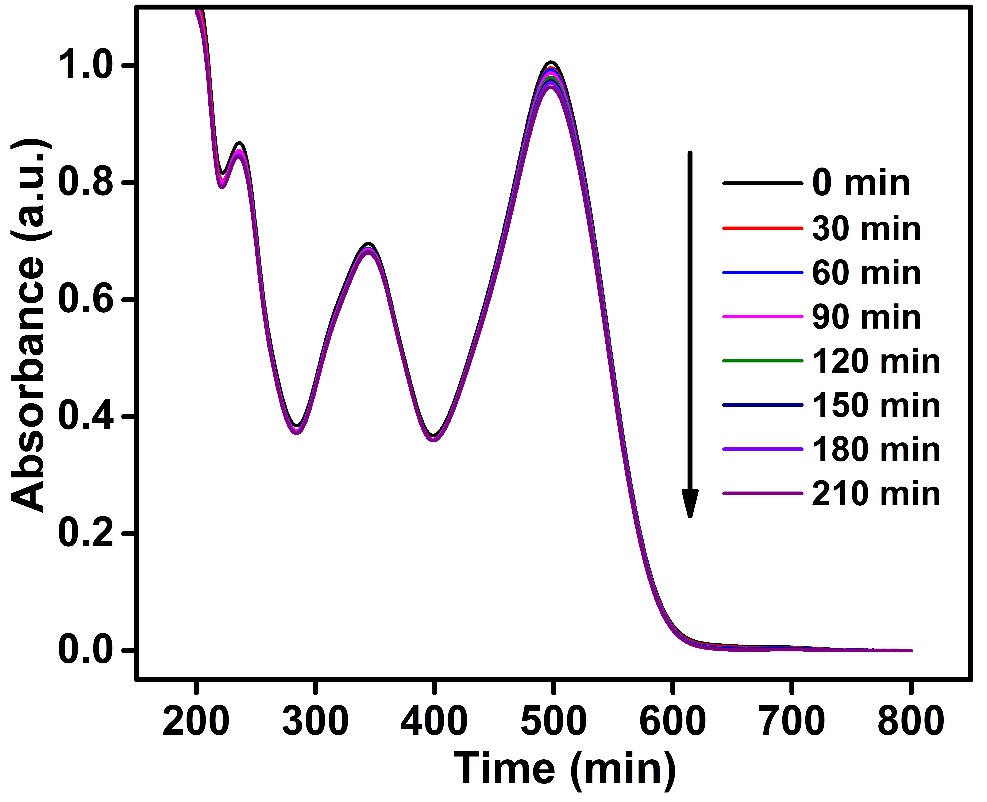


**Supplementary Figure 3.** Photolysis of congo red dye without Ag@ZnO NCs (photocatalyst).


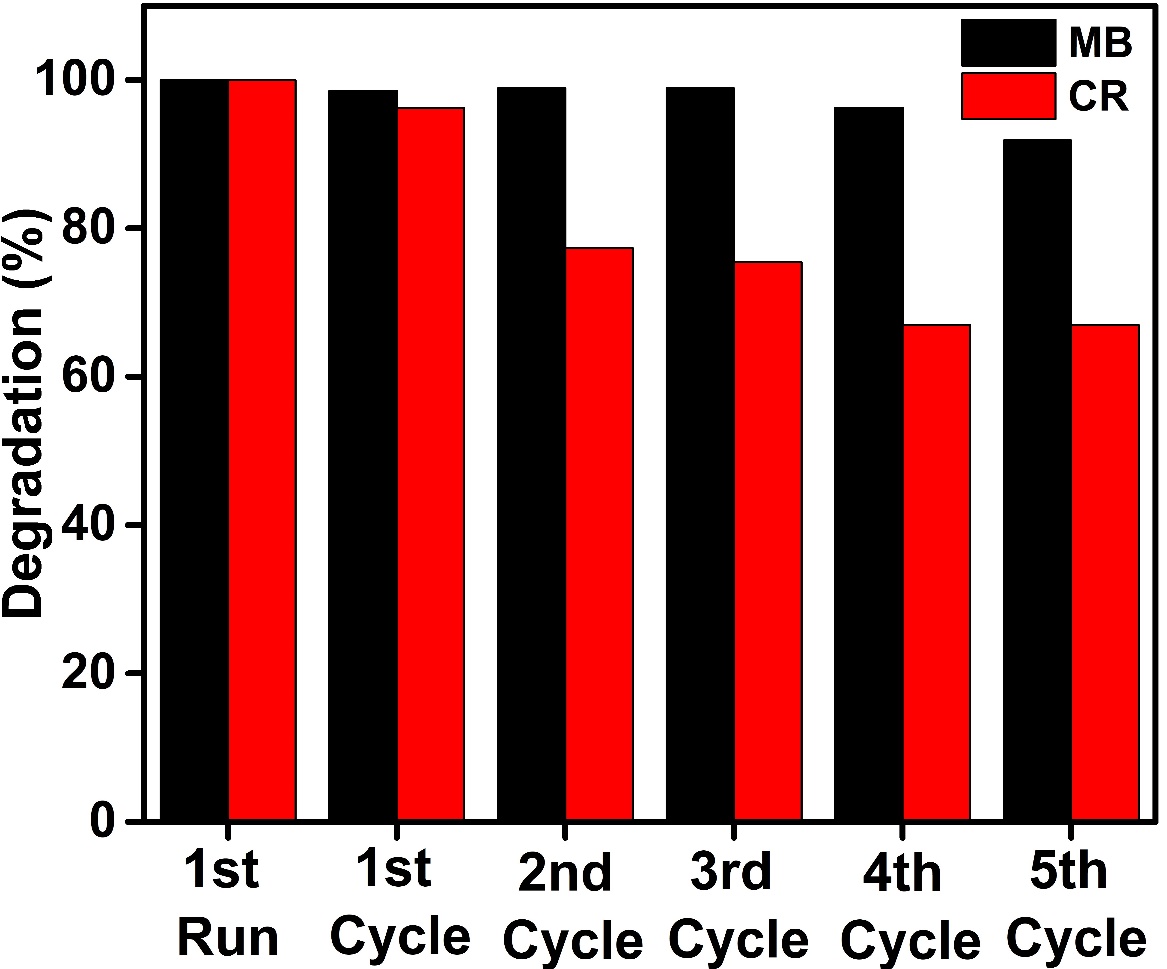


**Supplementary Figure 4.** Reusability of Ag_0.2_@ZnO NC as photocatalysts in the degradation of MB and CR up to five cycles.
